# Causality of genetically determined glucosamine supplementation on cognition and sarcopenia: a Mendelian randomization study

**DOI:** 10.3389/fendo.2024.1404308

**Published:** 2024-12-23

**Authors:** Yi Kang, Yidan Tang, Weishuang Kong, Tao Zhu, Guo Chen

**Affiliations:** ^1^ Department of Anesthesiology, West China Hospital, Sichuan University, Chengdu, China; ^2^ Laboratory of Anesthesia and Critical Care Medicine, National-Local Joint Engineering Research Centre of Translational Medicine of Anesthesiology, West China Hospital, Sichuan University, Chengdu, China; ^3^ Department of Surgery, Xuanwei Hospital of Traditional Chinese Medicine, Xuanwei, China

**Keywords:** Mendelian, glucosamine, cognition, sarcopenia, CRP, BMR, causality

## Abstract

**Background:**

Evidence indicates a negative link between glucosamine and age-related cognitive decline and sarcopenia. However, the causal relationship remains uncertain. This study aims to verify whether glucosamine is causally associated with cognitive function and sarcopenia.

**Method:**

Forty-eight genetic variants linked to glucosamine use were extracted from the MRC-IEU consortium. Besides, we gathered cognition proxy indicators [cognitive performance and fluid intelligence score (FIS)], and sarcopenia-related indicators, namely, appendicular lean mass (ALM), whole body fat-free mass (WBFM), low hand grip strength, facial aging (FA), moderate to vigorous physical activity levels, usual walking pace and DNA methylation GrimAge acceleration from the large publicly available genome-wide association studies. Initially, Mendelian randomization (MR) analyses were conducted to ascertain the causal impact of glucosamine on cognition and sarcopenia-related traits. Subsequently, the two-step MR and multivariable MR (MVMR) were employed to examine whether any mediators causally mediated the observed associations.

**Result:**

MR analysis indicated that glucosamine was associated with increased cognitive performance (*p* = 8.46E-04), FIS (*p* = 7.50E-04), ALM (*p* = 6.45E-08), WBFM (*p* = 1.97E-03), usual walking pace (*p* = 2.55E-07), and moderate to vigorous physical activity levels (*p* = 3.29E-03), but associated with decreased FA risk (*p* = 3.77E-05) and DNA methylation GrimAge acceleration (*p* = 0.001). However, there were no significant causal associations between glucosamine and low hand grip strength. Multivariable MR showed that glucosamine continued to have a significant effect on cognitive performance, FIS, ALM, WBFM, usual walking pace, and moderate to vigorous physical activity levels after controlling for osteoarthritis (OA) and body mass index (BMI) (*p* < 0.05). We further found that C-reactive protein levels (CRP) may mediate the association of glucosamine and ALM, WBFM, usual walking pace, and physical activity (p < 0.05), and basal metabolic rate (BMR) may mediate the association of glucosamine and cognitive performance, FIS, ALM, WBFM, and usual walking pace (*p* < 0.05).

**Conclusion:**

Regular glucosamine use enhances cognitive function and postpones sarcopenia for preserving the functional capacities necessary, and the impact of glucosamine on cognition and sarcopenia could be partially attributed to the mediation of BMR and CRP.

## 1 Introduction

The phenomenon of aging societies is becoming a prevalent worldwide trend. Epidemiological studies indicate that now, 11% of the global population is aged 60 or over. Projections suggest that by the year 2050, this percentage will double to 22% of the population (1). Notably, the prevalence of aging populations has resulted in the growth of certain chronic age-related ailments, such as cognitive decline, neurodegenerative changes, and sarcopenia, even among older adults who do not develop dementia (2). Sarcopenia, characterized by the gradual decrease in muscle mass and strength during the aging process, is strongly associated with various adverse outcomes, including falls, frailty, mortality, and worse cognitive impairment, significantly impacting overall health, reducing locomotion economy and ease (3–5). Furthermore, sarcopenia and cognitive decline exhibit a shared pathophysiological pathway, including the muscle-brain Axis (6, 7). Due to the lack of effective pharmacological interventions for these social and public health issues, there has been a significant focus on thoroughly investigating potentially modifiable protective factors.

Glucosamine, classified as an amino sugar, is a naturally derived compound that promotes the synthesis of glycosaminoglycans. As a non-vitamin, non-mineral dietary supplement, glucosamine gained popularity as one of the most commonly used complementary health approaches in 2012, and its usage has steadily increased over the past 15 years with minimal reported adverse effects. Approximately 15.9% of middle-aged Australian women and 22.0% of participants aged 45 and over are estimated to utilize glucosamine (8, 9). Glucosamine and its derivatives are among the most commonly recommended over-the-counter (OTC) supplements by physicians for the treatment of osteoarthritis (OA) (10). However, the clinical efficacy and long-term advantages of these supplements remain subjects of ongoing debate. Moreover, studies have demonstrated that glucosamine consumption may manifest a diverse array of biochemical and pharmacological functions, with many of its applications relying on its anti-inflammatory and antioxidant properties, which encompass anticancer activity, cardiovascular effects, cognitive effects, neuroprotective effects, and more (11–15). Therefore, several studies suggest that regular glucosamine supplementation may confer a protective effect against mortality in the general population and mitigate age-related cognitive decline, potentially extending their overall health span (16, 17). Nevertheless, the appropriateness of using glucosamine as a preventive medication for the elderly remains uncertain. Moreover, study data have pointed to increased life expectancy and decreased incident vascular dementia in individuals who are supplemented with glucosamine (18, 19). However, the causal association between glucosamine and cognition and sarcopenia has yet to be demonstrated.

Due to limitations such as residual confounding, potential reverse causality, inadequate adjustment, and a focus on correlation rather than causation, observational studies have been hindered in identifying a causal effect of glucosamine supplementation on cognition and sarcopenia. On the other hand, the implementation of large-scale randomized controlled trials (RCTs) poses challenges. Utilizing genetic variants as proxies for glucosamine, mendelian randomization (MR) circumvents several limitations and offers genetic evidence supporting causal associations (20). We performed the present MR study to evaluate the causality between glucosamine and changes in aging and cognition by analyzing the summary-level genome-wide association studies (GWAS) data of glucosamine, cognition proxy indicators as well as aging and sarcopenia-related indicators such as cognitive performance, facial aging, appendicular lean mass (ALM), whole body fat-free mass (WBFM), grip strength, DNA methylation GrimAge acceleration, usual walking pace and other relevant traits. Therefore, we hypothesize that regular glucosamine use may causally impact cognition and sarcopenia-related traits.

## 2 Materials and methods

### 2.1 Study design

A two-sample MR was utilized to assess the causal association of glucosamine on cognition and sarcopenia-related traits. Briefly, glucosamine served as the exposures, while two indicators of cognition and seven sarcopenia-related traits served as the outcomes. This two-sample MR study consisted of two phases of analysis. In the first phase, we first examined the causal effects of glucosamine on 2 cognitive traits and 7 sarcopenia-related traits. Then, we investigated whether these causal effects were independent of OA and BMI. In the second phase, we assessed the mediating roles of CRP and BMR in the causal associations between glucosamine and cognitive function outcomes and sarcopenia-related traits. MR was performed under three crucial assumptions (1). genetic IVs are associated with glucosamine (the relevance assumption) (2); genetic IVs are independent of confounding variables (the independence assumption) (3); genetic IVs only affect outcomes above through glucosamine (the exclusion restriction assumption) (21).

**Figure 1:**
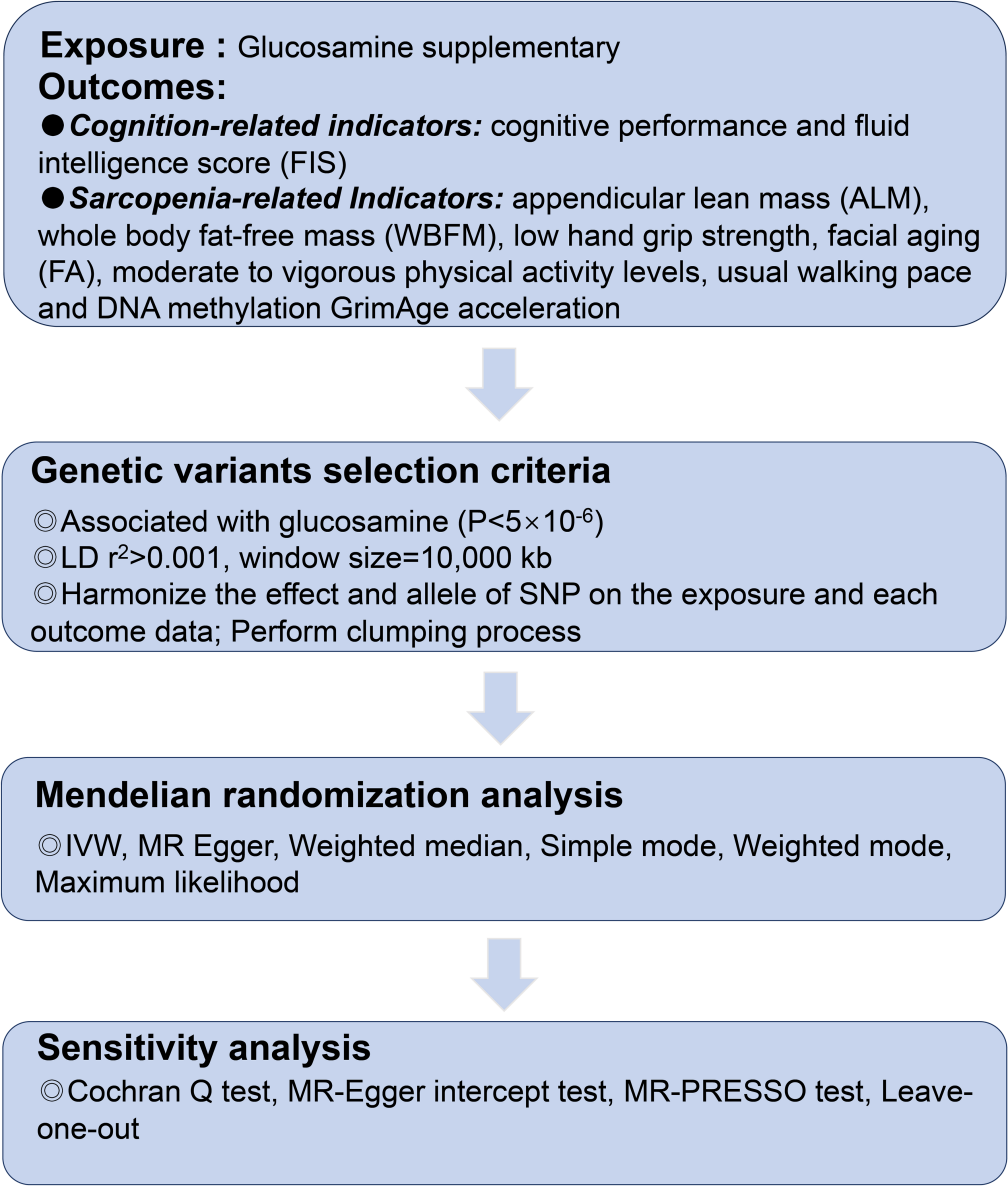
The flowchart of the study.

**Figure 2:**
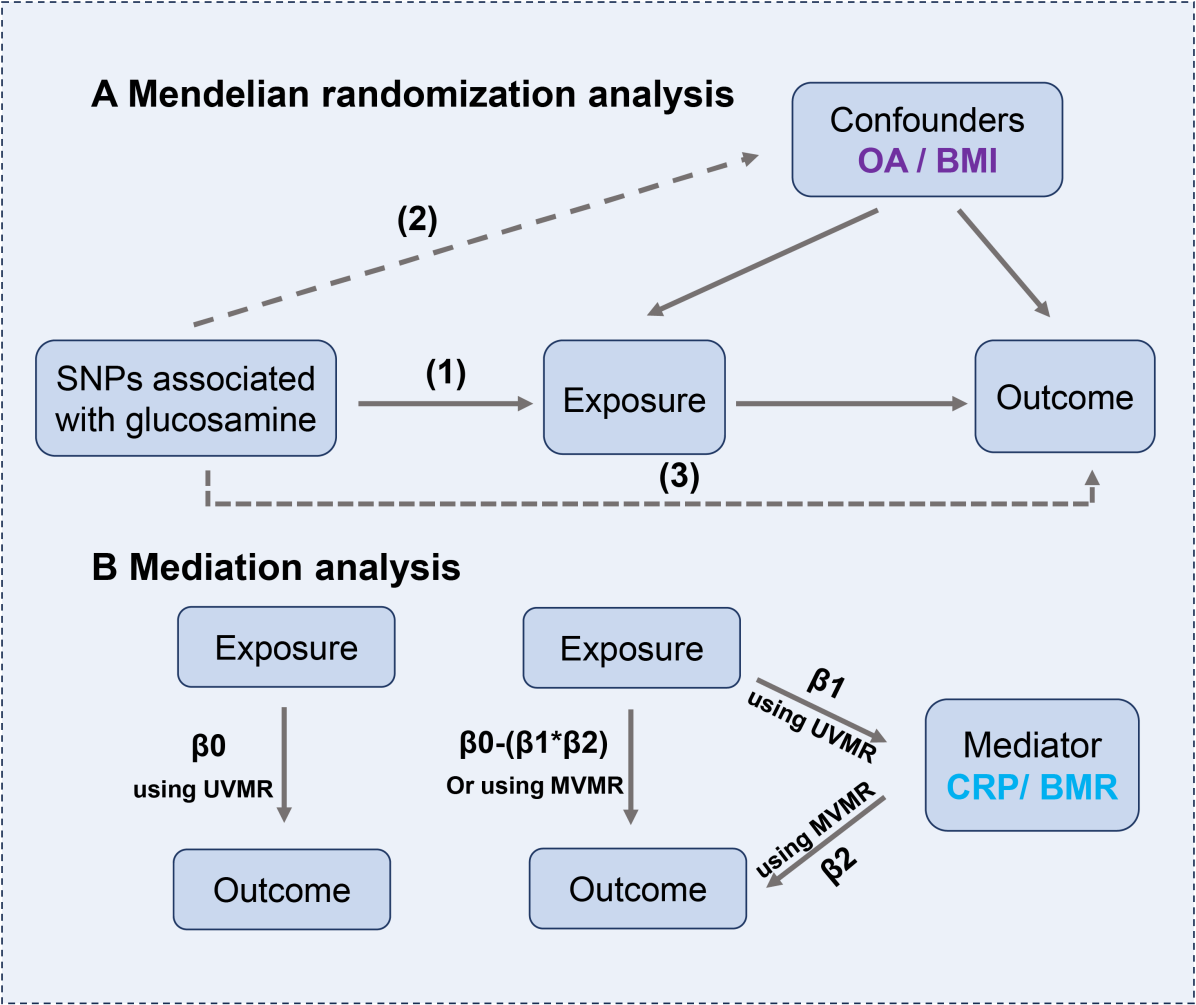
**(A)** The principles for mendelian randomization study are as follows: (1) genetic instrumental variables are associated with the exposure, (2) genetic instrumental variables are independent of confounding variables, (3) genetic instrumental variables only affect outcome through exposure. **(B)** Mediation analysis. The entire effect (β0) of glucosamine on outcomes can be split into direct (β0-β1*β2) and indirect effects (β1*β2).

This study was reported in accordance with the Strengthening the Reporting of Observational Studies in Epidemiology using Mendelian Randomization (STROBE-MR) (22). Summary statistics were obtained from the reputable publicly available GWAS conducted by European participants. Initially, mendelian randomization (MR) analyses. The included research and databases obtained the ethical committee’s approval and informed consent from participants. The GWAS datasets utilized in this investigation are detailed in **Supplementary Table 1**. The ethical approval for the GWAS studies can be found in the referenced studies provided below. As instrumental variables (IVs), single-nucleotide polymorphisms (SNPs) strongly associated with glucosamine at genome-wide significance were selected (*p* < 5 ×10^-6^). The flowchart is shown in **Figure 1** and **Figure 2**.

### 2.2 Data source and selection of genetic instruments

Genetic variants significantly associated with glucosamine were extracted from the largest Open GWAS data of the Medical Research Council Integrative Epidemiology Unit (MRC-IEU) consortium, which comprised 89,339 cases and 372,045 controls, available through the UK Biobank (https://gwas.mrcieu.ac.uk/datasets/ukb-b-11535/). A baseline touchscreen questionnaire was used to acquire habitual glucosamine information. The participants were asked, “Do you regularly take any of the following?” The touchscreen questionnaire allowed participants to pick various answers from a variety of **Supplementary Materials** (Mineral and other dietary supplements). We classified glucosamine users as follows: 0 no; 1 yes.

Genetic variants associated with glucosamine at genome-wide significance (*p* < 5×10^-6)^ were identified and extracted as potential IVs. Then, we performed the clumping procedure (linkage disequilibrium r2 < 0.001 within kb = 10,000) to ensure that genetic variants were independent of each other. To assess weak instrumental bias, the F statistics were calculated using the following formula: F = R^2^ (N–k–1)/[(1–R^2^) k], where N represents the sample size and k denotes the number of included SNPs, R^2^ represents the proportion of variability that can be attributed to each SNP. Besides, R^2^ can be calculated by the formula: R^2^ = 2 × β^2^ × EAF × (1 – EAF), where β indicates the estimated effect size of the IVs and EAF represents the effect allele frequency. A genetic variation used as a weak IV is considered when the F-statistic is less than 10, as it can potentially introduce bias to the results. Details of GWAS studies are provided in the **Supplementary Table 1**.

### 2.3 Data source for cognition and sarcopenia-related traits

Cognition outcomes included cognitive performance and fluid intelligence score (FIS) traits. Cognitive performance data were extracted from the largest publicly available GWAS in 257,841 European-ancestry participants from COGENT Consortium (35 sub-cohorts) and UK Biobank, with adjustment for sex, age, and population stratification (23). GWAS data for FIS data were from the Medical Research Council Integrative Epidemiology Unit (MRC-IEU) consortium, available through the UK Biobank. Seven traits were selected for valid predictors of sarcopenia: appendicular lean mass (ALM), whole body fat-free mass (WBFM), low hand grip strength, facial aging (FA), moderate to vigorous physical activity levels, usual walking pace and DNA methylation GrimAge acceleration (24). Among them, grip strength was used to measure muscle strength, and WBFM and ALM were used to measure muscle mass. ALM is predominantly determined by skeletal muscle and has been shown to make up ≥75% of skeletal muscle in the body, whereas WBFM comprises smooth muscle, cardiac muscle, and skeletal muscle (25). The GWAS meta-analysis of 256,523 Europeans aged 60 years and older from 22 cohorts was utilized to extract grip strength data; the criteria for this analysis were established by the European Working Group on Sarcopenia in Older People (EWGSOP) consortium (grip strength <30 kg Male; <20 kg Female) (26).

FA serves as a prominently visible indicator of aging. The summary statistics for facial aging of GWAS were obtained from the UK Biobank, which contained a total of 423,999 participants of European ancestry (194,391 males and 229,601 females, aged 40–69). Facial aging (FA) is evaluated with non-subjective perceived age through a touchscreen questionnaire. 103,300 individuals reported appearing to be their age. A total of 312,062 individuals reported appearing younger than their biological age, while 8,630 individuals reported appearing older than their biological age. The participants were asked, “Do people say that you look?” and allowed to pick the answer from a variety of **Supplementary Materials**. The researchers coded the participants in accordance with their actual age and perceived age. Participants were assigned codes as follows: 0 indicated that they appeared older, 1 stated that they appeared younger, and 0.5 indicated that they appeared to be their age (third-party observations, including those of non-participants and non-researchers, were conducted without the knowledge of the participant’s actual ages). The summary statistics for moderate to vigorous physical activity levels were derived from a GWAS conducted on 377,234 UK Biobank participants (27). Just as low physical performance is one of the characteristics of sarcopenia, clinical walking speed is a fast, safe, and highly reliable test for sarcopenia. Usual walking pace data were obtained from the UK Biobank with 459,915 European individuals. DNA GrimAge acceleration is a measure of accelerated biological aging. It is established by combining chronological age, sex, and surrogate biomarkers based on DNA methylation for seven plasma proteins and smoking pack-years. The data for DNA methylation GrimAge acceleration was derived from McCartney et al. GWAS study with 34,467 subjects (28). Details of GWAS studies are provided in the **Supplementary Table 1**.

### 2.4 Data source for mediators

Oral glucosamine has traditionally been advised for managing knee and hip OA (29). Therefore, OA was selected and identified as a major confounder. Summary statistics for OA were obtained from the largest publicly available GWAS with 484,598 participants (39,515 OA cases and 445,083 controls), which utilized the UK Biobank data (30). We also assessed the effect of body mass index (BMI) as a confounder. BMI data were derived from the largest publicly available GWAS by the Genetic Investigation of Anthropometric Traits (GIANT) consortium, which included 681,275 European individuals (31). C-reactive protein (CRP) levels may be involved in altered cognitive function and accelerated aging. Besides, the development of sarcopenia usually brings about a decrease in the basic metabolic rate (BMR) of the organism and a decrease in trunk activity. Therefore, CRP and BMR were selected and identified as the mediators in the effect of glucosamine on cognition and sarcopenia‐related traits. CRP data were obtained from the largest publicly available GWAS with 575,531 participants of European ancestry, which was conducted in the UK Biobank participants (427,367 European descent) and the Cohorts for Heart and Aging Research in Genomic Epidemiology (CHARGE) Consortium (575,531 European descent) (32). Summary statistics for basal metabolic rate were derived from the MRC-IEU consortium, available through the UK Biobank, which included 454,874 European participants. Details of GWAS studies are provided in the **Supplementary Table 1**.

### 2.5 Statistical analysis

MR estimates were reported as odds ratios (ORs) with 95% confidence intervals (CIs). We evaluated the causal relationship of glucosamine with two cognition traits and seven sarcopenia-related traits by applying univariable MR analysis (UVMR), with estimates represented as β0. Then, we applied multivariable MR (MVMR) to evaluate the causal effect of glucosamine on cognition and sarcopenia-related traits with both adjustments for OA and BMI to determine whether glucosamine was causally associated with the cognition and sarcopenia-related outcomes independent of OA and BMI.

We applied fixed effects inverse variance weighted (IVW-FE) as the major analysis for UVMR. To enhance the robustness of IVW-FE results, we also employed MR Egger, maximum likelihood, weighted median, and weighted mode methods for additional validation. The MR-Egger method aids evaluating horizontal pleiotropy and bias caused by weak instruments (33). A weighted median approach could effectively mitigate the impact of outlier SNPs on the results, thereby enhancing robustness (33). The weighted mode approach provides dependable estimates when the majority of similar individual-instrument causal effect estimates are derived from genuine instrumental variables, even if a large portion of them is invalid (33). Sensitivity analyses were conducted to assess potential biases and underlying assumptions in the MR analysis, aiming to validate and refine the results. Heterogeneity was assessed using the Cochran Q test. There is no heterogeneity if the p-value of Cochran’s Q test is greater than 0.05. Once heterogeneity (*p* < 0.05) has been identified, use the multiplicative random effects IVW (IVW-MRE) method to ascertain the causal influence. Besides, the MR pleiotropy residual sum and outlier (MR-PRESSO) test and MR-Egger were performed to assess possible horizontal pleiotropy. Horizontal pleiotropy is described as some instruments’ additional biological effects that influence the outcome independently of the exposure. The presence of horizontal pleiotropy can be detected using the MR-PRESSO global test, while the MR-PRESSO outlier test is utilized to exclude outlier SNPs and assesses the impact of excluding these SNPs on causal estimations (34). The MR-Egger intercept test is additionally employed to assess the horizontal pleiotropy (35). A leave-one-out method was employed to identify high-influence points and whether a particular SNP influenced the significant results.

Furthermore, the confounding analysis utilized Phenoscanner V2 (http://www.phenoscanner.medschl.cam.ac.uk/) (36). We have identified certain diseases or physical ailments that correlate highly with IVs at a significance level of *p* < 5 × 10^-6^. Subsequently, we condensed and examined pertinent data pertaining to IVS, GWASes, and illnesses. This approach not only assists in identifying important factors that need to be accounted for in MVMR, but it also enables us to explore the process of mediation and potential causal pathways.

MVMR provides insights into the extent to which the mediator explains the relationship between the exposure and the outcome (21). In addition, MVMR allows for the simultaneous examination of exposure while considering potential confounding factors. The IVW method was used for the MVMR. Moreover, we performed two‐step MR to explore whether CRP levels and BMR mediate the causal associations between glucosamine and cognition and sarcopenia‐related outcomes. The first step was to use UVMR to estimate the causal effect of glucosamine on mediators (CRP and BMR, separately), with each estimate represented as β1. The second step was to estimate the causal effect of the mediators on outcomes (cognition and sarcopenia‐related traits), with adjustment for glucosamine using MVMR (MVMR estimate represented as β2). The entire effect of glucosamine on outcomes can be split into direct (not mediated by mediators) and indirect effects (effect mediated by the mediators). The following conditions exist: 1) If the coefficients β0, β1, and β2 are all statistically significant, it suggests that a causal relationship between the exposure and outcome variables. Furthermore, this relationship may be influenced to some extent by mediating factors. The mediation proportion of mediator (CRP and BMR) in the causal association between glucosamine and outcomes was calculated as the product of β1 and β2 divided by β0; 2) If the coefficient β0 is not statistically significant, yet the coefficients β1 and β2 are both statistically significant, it suggests that the mediator variable mainly influences the impact; 3) If the coefficient β0 is statistically significant and either β1 or β2 is not statistically significant, then indicates that the mediating effect does not play a role in mediating the relationship between exposure and the result (37).

A significance level of 0.05 was implemented for the *p*-value. To adjust for multiple testing (several exposures), the MR effect estimates were statistically significant at less than five percent using the Benjamini-Hochberg false discovery rate (FDR). All analyses were carried out using packages “TwoSampleMR,” “MendelianRandomization,” “MR-PRESSO,” and “Phenoscanner” in R version 4.3.1.

## 3 Result

### 3.1 Validity of instrumental variables

- Only 5 SNPs were significantly associated with glucosamine when at the threshold of *p* < 5 × 10^-8^. Therefore, forty-eight SNPs associated with glucosamine at genome-wide significance of *p* < 5 × 10^-6^ were identified and selected (**Supplementary Table 2**). The F statistic of all identified SNPs was over 10. The harmonies data for glucosamine and cognition and sarcopenia-related traits are shown in **Supplementary Table 3**.

### 3.2 Univariable MR analysis

#### 3.2.1 Effect of glucosamine on cognition

**Figure 3:**
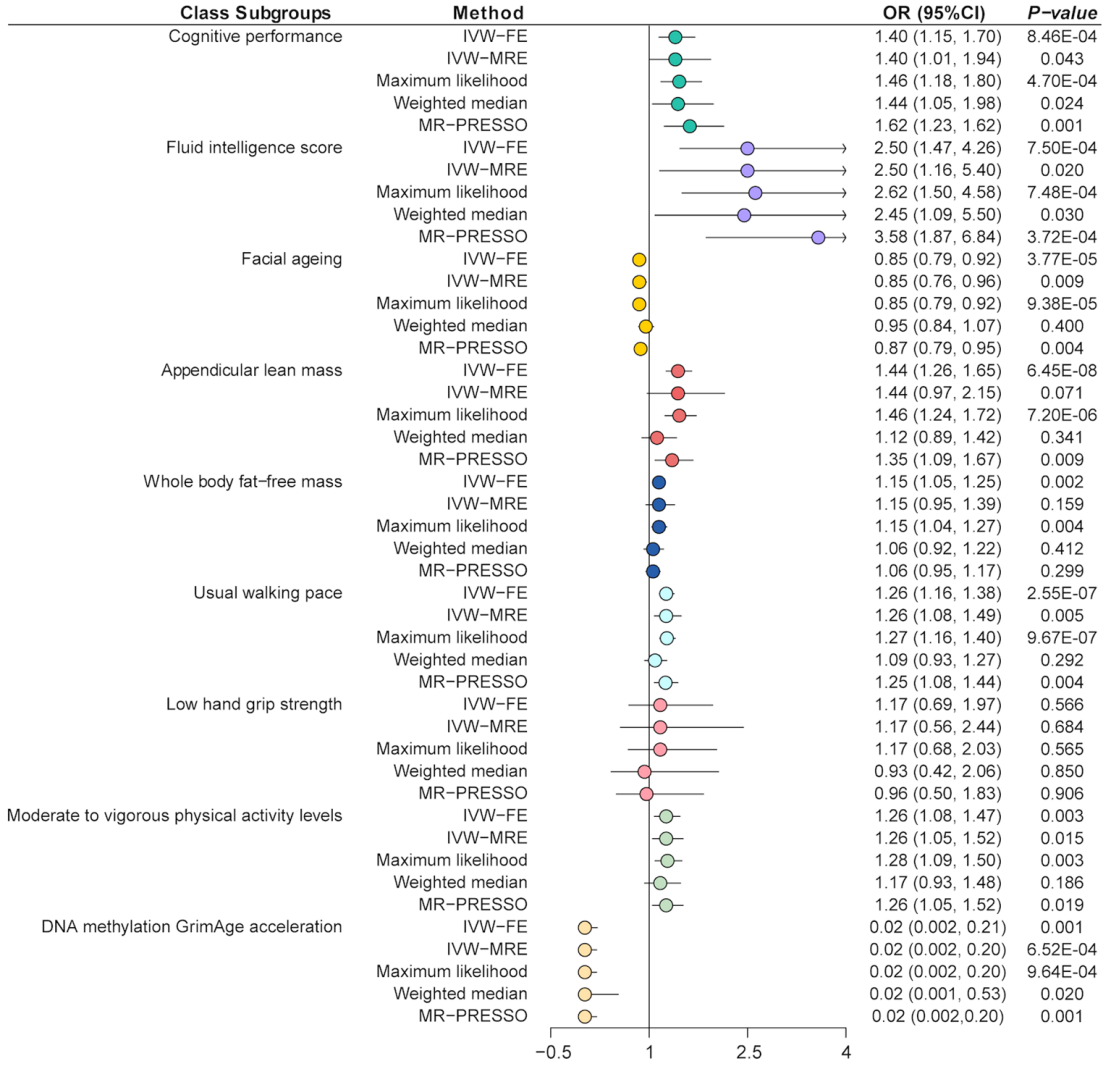
Mendelian randomization analysis of the effect of glucosamine on cognition and sarcopenia. IVW-FE, inverse variance weighted (fixed effects); IVW-MRE, inverse variance weighted (multiplicative random effects); MR-PRESSO, Mendelian randomization pleiotropy residual sum and outlier.

The main results of the MR analysis are shown in **Figure 3**. We performed an UVMR analysis to investigate the association between glucosamine and cognition traits. The main analytical method applied in the MR analysis, IVW-FE, discovered that taking glucosamine significantly increased cognitive performance (OR = 1.40, 95% CI = 1.15-1.70, *p* = 8.46E-04), and FIS (OR = 2.50, CI = 1.47-4.26, *p* = 7.50E-04) (**Figure 3**; **Supplementary Tables 4**, **5**). Besides, due to the detected heterogeneity by Cochran Q statistic (*p* < 0.01), we applied the IVW-MRE method and also detected consistent results (**Supplementary Tables 4**–**6**). Other sensitive analyses methods such as maximum likelihood and weighted median, also detected the same results (**Supplementary Tables 4**, **5**). The MR-Egger regression analysis showed that there was no horizontal pleiotropy (Egger intercept cognitive performance = -0.0002, *p* = 0.91; Egger intercept FIS = -0.001, *p* = 0.87) (**Supplementary Table 7**). Horizontal pleiotropy was shown by the results of the MR-PRESSO analysis, which also identified some outliers (**Supplementary Table 8**). The results also indicated that, after removing this SNP, trends of recalculated results are consistent with the initial results (IVW cognitive performance, OR = 1.62, 95% CI = 1.23-2.14, *p* = 1.29E-03; IVW FIS, OR = 3.58, 95% CI = 1.87-6.84, *p* = 3.72E-04). Further leave-one-out tests suggested that the associations between glucosamine and FIS were reliable, while the association between glucosamine and cognitive performance was less robust (**Supplementary Figure 1**).

#### 3.2.2 Effect of glucosamine on sarcopenia-related traits

The IVW-FE method showed that glucosamine was associated with increased ALM (OR = 1.44, 95% CI = 1.26-1.65, *p* = 6.45E-08), WBFM (OR = 1.15, 95% CI = 1.05-1.25, *p* = 1.97E-03), usual walking pace (OR = 1.26, 95% CI = 1.16-1.38, *p* = 2.55E-07) as well as moderate to vigorous physical activity levels (OR = 1.26, 95% CI = 1.08-1.47, *p* = 3.29E-03), but associated with decreased FA risk (OR = 0.85, 95% CI = 0.79-0.92, *p* = 3.77E-05) and decreased DNA methylation GrimAge acceleration (OR = 0.02, 95% CI = 0.002-0.21, *p* = 0.001) (**Figure 3**; **Supplementary Tables 4**, **5**). Besides, there were no significant causal associations between glucosamine and low hand grip strength (OR = 1.17, 95% CI = 0.69-1.97, *p* = 0.56). Besides, due to the detected heterogeneity by Cochran Q statistic (*p* < 0.01), the IVW-MRE method also detected the consistently effective effect of glucosamine on FA, physical activity, and usual walking pace (**Supplementary Tables 4**–**6**). The MR-Egger regression analysis showed that there was no horizontal pleiotropy except between glucosamine and physical activity (Egger intercept = -0.003, *p* = 0.03) (**Supplementary Table 7**). Horizontal pleiotropy was shown by the results of the MR-PRESSO analysis, which also identified some outliers (**Supplementary Table 7**). The results also indicated that, after removing this SNP, trends of recalculated results are consistent with the initial results. Further leave-one-out tests suggested that the associations were reliable, while the association between glucosamine and ALM was less robust (**Supplementary Figure 2**).

### 3.3 Confounding analysis

**Figure 4:**
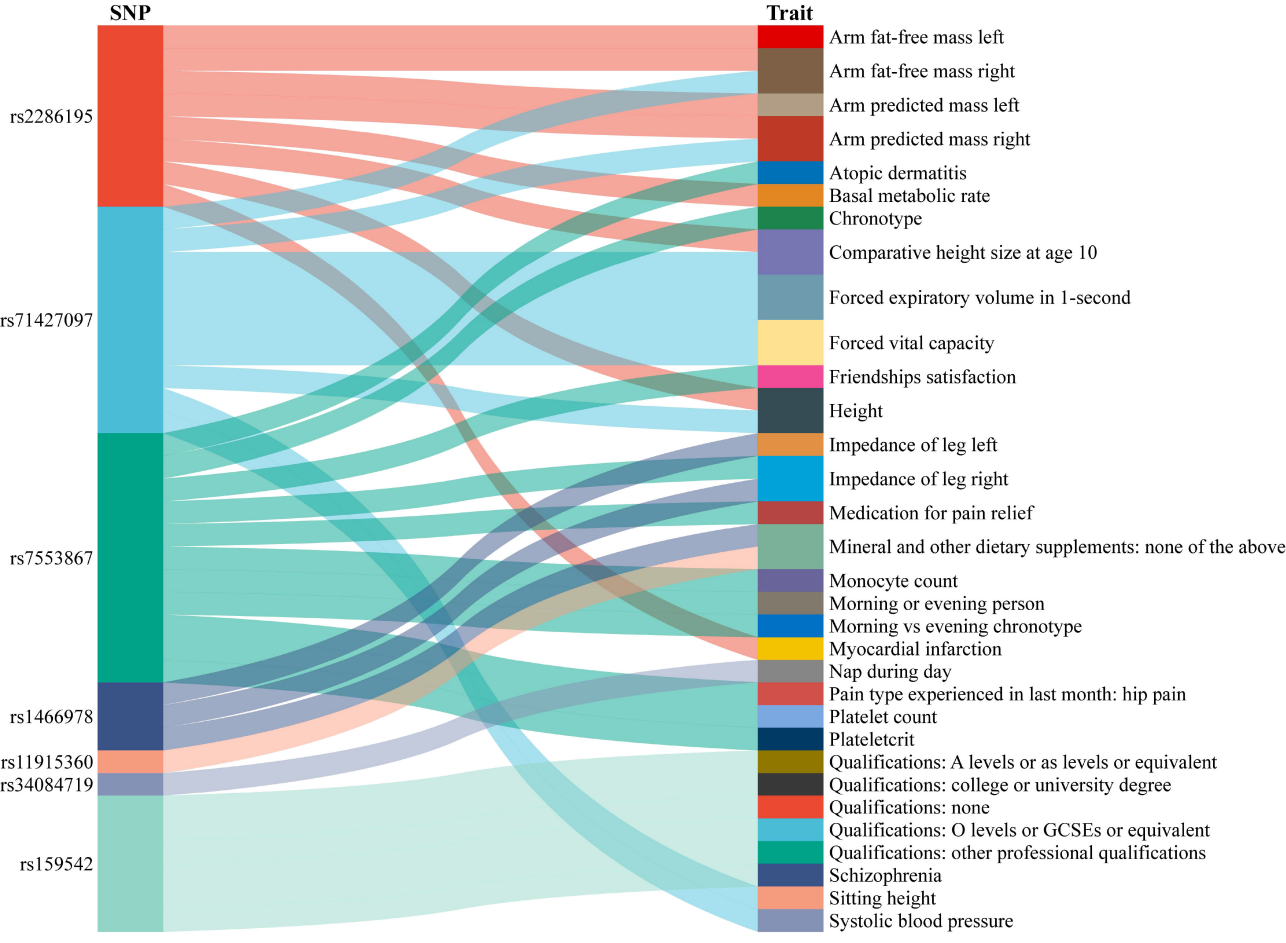
The result of confounding analysis. SNP, single nucleotide polymorphism.

After analyzing and evaluating the related information about glucosamine-associated SNPs, GWASes, and Diseases through Phenoscanner, we found some potential confounders or outcomes, mainly including arm fat-free mass, arm predicted mass, chronotype, morning or evening person, forced expiratory volume in 1-second, forced vital capacity, and qualifications, etc. (**Figure 4**).

### 3.4 Multivariable MR analysis

We performed MVMR Analysis by both adjusted OA and BMI to further assess the associations between glucosamine and outcomes. After adjusting both OA and BMI, we found that taking glucosamine continued to have a significant effect on cognitive performance (OR = 1.87, 95% CI = 1.23-2.83, *p* = 3.42E-03), FIS (OR = 4.88, CI = 1.87-12.73, *p* = 1.18E-03), ALM (OR = 3.59, 95% CI = 2.29-5.63, *p* = 2.66E-08), WBFM (OR = 1.66, 95% CI = 1.33-2.08, *p* = 7.38E-06), usual walking pace (OR = 1.53, 95% CI = 1.34-1.76, *p* = 9.46E-10), and moderate to vigorous physical activity levels (OR = 1.40, 95% CI = 1.12-1.74, *p* = 3.25E-03) (**Supplementary Table 9**).

### 3.5 Mediation analysis

We further explored the mediating roles of CRP and BMR in the improvement of cognition and sarcopenia‐related traits by glucosamine using MVMR coupled with a two-step MR method, respectively (**Supplementary Table 10**). The protective effects of glucosamine on ALM, WBFM, usual walking pace, and moderate to vigorous physical activity levels were continued after adjusting for CRP. However, the effects of glucosamine on cognitive performance, FIS, FA, DNA methylation GrimAge acceleration, and low hand grip strength were weakening after adjusting for CRP, suggesting this association was largely affected by CRP. The proportion mediated by CRP in the associations between glucosamine and outcomes was 4% for ALM, 1.4% for WBFM, 5% for usual walking pace, 3% for moderate to vigorous physical activity levels (**Table 1**).

**Table 1:** The mediation effect of glucosamine on cognition, and sarcopenia-related traits via CRP.

	Effect of glucosamine on cognition, and sarcopenia-related traits after adjusted by CRP		Effect of CRP on cognition, and sarcopenia-related traits	
Outcomes	Effect estimate (IVW): OR (95% CI)	*p-value*	Effect estimate (IVW): OR (95% CI)	*p-value*
cognitive performance	2.07 (1.36, 3.16)	7.31E-04	0.98 (0.96, 1.01)	0.210
FIS	4.49 (1.65, 12.24)	0.003	0.94 (0.89, 1.004)	0.068
ALM	3.98 (2.28, 6.95)	1.21E-06	0.93 (0.89, 0.96)	1.15E-05
WBFM	1.84 (1.21, 2.79)	0.004	1.03 (1, 1.06)	0.025
Usual walking pace	1.46 (1.2, 1.77)	1.47E-04	0.95 (0.93, 0.96)	1.81E-19
Moderate to vigorous physical activity levels	1.33 (1.06, 1.67)	1.49E-02	0.96 (0.95, 0.98)	8.58E-08
DNA methylation GrimAge acceleration	0.18 (0.01, 2.43)	0.197	1.44 (1.22, 1.69)	9.13E-06

Moreover, the protective effects of glucosamine on cognitive performance, ALM, WBFM, usual walking pace, and moderate to vigorous physical activity levels were continued after adjusting for BMR. However, the effects of glucosamine on FIS, FA, and DNA methylation GrimAge acceleration were weakening after adjusting for BMR, suggesting this association was largely affected by BMR. Specifically, the proportion mediated by BMR in the associations between glucosamine and outcomes was and 3% for cognitive performance, 3% for FIS, 39% for ALM, 71% for WBFM, and 3% for usual walking pace (**Table 2**).

**Table 2:** The mediation effect of glucosamine on cognition, and sarcopenia-related traits via BMR.

	Effect of glucosamine on cognition, and sarcopenia-related traits after adjusted by BMR		Effect of BMR on cognition, and sarcopenia-related traits	
Outcomes	Effect estimate (IVW): OR (95% CI)	*p-value*	Effect estimate (IVW): OR (95% CI)	*p-value*
cognitive performance	1.5 (1.04, 2.17)	0.031	1.08 (1.04, 1.13)	4.24E-05
FIS	2.3 (0.98, 5.41)	0.057	1.23 (1.13, 1.35)	4.74E-06
ALM	1.71 (1.14, 2.56)	9.55E-03	3.69 (3.54, 3.85)	< 0.001
WBFM	1.05 (1.01, 1.1)	0.026	2.63 (2.62, 2.65)	< 0.001
FA	0.92 (0.82, 1.03)	0.169	1.07 (1.06, 1.08)	3.97E-27
Usual walking pace	1.34 (1.14, 1.57)	3.12E-04	0.93 (0.92, 0.95)	9.34E-17
Moderate to vigorous physical activity levels	1.43 (1.00, 2.06)	0.051	0.94 (0.91, 0.98)	0.001
DNA methylation GrimAge acceleration	0.12 (0.01, 1.16)	0.067	1.53 (1.21, 1.94)	3.72E-04

## 4 Discussion

In this research, we primarily used large-sample GWAS data to assess the connections between glucosamine and cognition as well as sarcopenia proxy indicators by MR analysis. We conducted various MR analysis methods, including UVMR, MVMR, and mediation analysis, which showed that (1) glucosamine use is probably associated with enhanced cognitive performance, FIS, ALM, WBFM, usual walking pace and physical activity, and decreased risk of FA and DNA methylation GrimAge acceleration; (2) this protective effect for glucosamine on cognitive performance, FIS, ALM, WBFM, usual walking pace, and physical activity remain even after adjusting for both OA and BMI; (3) CRP may mediate the association of glucosamine and ALM, WBFM, usual walking pace, and physical activity; (4) BMR may mediate the association of glucosamine and cognitive performance, FIS, ALM, WBFM, and usual walking pace.

Several studies have investigated the longitudinal relationship between habitual glucosamine administration and cognitive impairment in the general population, which is consistent with our results. Glucosamine is a suggested over-the-counter medicine for adults with osteoarthritis. It has been in widespread usage since 2012. Relevant cohort studies and analyses offer information on the utilization of glucosamine for cognitive impairment. Some clinical studies have shown a correlation between the intake of glucosamine and the safeguarding of cognitive function. A large-scale population-based cohort study with baseline data from 502 647 participants aged 37 to 73 years collected at 21 assessment centers from 2006 to 2010 to identify compounds associated with cognitive performance found that glucosamine improved cognitive function (38). Zhou et al. examined the long-term relationship between regular glucosamine supplementation and the likelihood of developing dementia related to certain causes. The study encompassed a total of 214,945 persons aged 60 and above, who possessed data regarding their usage of glucosamine and were free from dementia at the beginning of the study in the UK Biobank. During a 12-year period, the regular usage of glucosamine was found to be strongly linked to a reduced chance of developing vascular dementia. The study found that regular use of glucosamine supplements was substantially linked to a reduced risk of developing vascular dementia in older individuals, regardless of their APOE genotypes and baseline cognitive ability (19). Besides, a recent study conducted a comprehensive analysis using large-scale observations and two-sample Mendelian randomization (MR) to investigate the correlation between regular glucosamine use and the likelihood of acquiring dementia, including different subtypes of dementia. The study examined the risk of all-cause dementia, Alzheimer’s disease (AD), and vascular dementia between users and non-users of glucosamine through Cox proportional hazards modelling. A total of 494,814 people were included, and at baseline, 94,259 (19.0%) reported taking glucosamine. During a median follow-up of 8.9 years (IQR 8.3-9.7 years), this extensive study involving a large population found that regular use of glucosamine was linked to a 15% decrease in the likelihood of developing all-cause dementia, a 17% decrease in the likelihood of attention deficit disorder, and a 26% decrease in the likelihood of vascular dementia. Through MR analysis, Zheng et al. once again demonstrated a beneficial causative impact of regular glucosamine treatment on the risk of dementia (39). The aforementioned research provides valuable insights into the beneficial impact of glucosamine on cognitive function.

Glucosamine may prevent bone loss in aging and adjust bone turnover (40). A double-blinded, randomized controlled trial revealed that the administration of glucosamine during a 12-week strength-training program enhanced maximal muscle strength gain in patients with OA compared to treatment with a placebo (41). Besides, a randomized, double-blind, placebo-controlled study in Japan investigated that consumption of a combination of milk-fat globule membrane and glucosamine may improve joint function and physical performance (42). Cerbo et al. study reported that the dietary supplement, including glucosamine, could improve facial photoaging and skin sebum, hydration, and tonicity, which may be by modulating serum fibronectin, neutrophil elastase 2, hyaluronic acid, and carbonylated proteins (43). These results are consistent with ours, as we found that genetically predicted glucosamine use is causally associated with enhanced ALM, WBFM, usual walking pace and physical activity, and decreased risk of FA. In addition, a recent MR study found that lifelong higher levels of glucosamine may increase life expectancy (18). DNA methylation GrimAge acceleration outperforms other DNA methylation-based biomarkers regarding various health-related metrics. It is especially effective in predicting time-to-death and the time it takes for coronary heart disease to develop in relation to age (44). Here, we found that glucosamine use is causally associated with reduced DNA methylation GrimAge acceleration risk.

There is a high overlap between individuals taking glucosamine and those with OA (45). Besides, we conducted a MVMR by summarizing and assessing the SNPs linked with glucosamine, as well as the GWASes and diseases related to it (21). Through this process, we identified possible confounders and included them in our study. Therefore, we consider OA and BMI as potential confounding variables and include them in the multivariate analysis. The MVMR analysis revealed a robust causal effect of glucosamine on cognitive performance, FIS, ALM, WBFM, usual walking pace, and physical activity. In addition, the progression of aging and the development of sarcopenia are strongly associated with elevated levels of CRP and a reduced BMR (46–48). A significant inverse association was observed between glucosamine and CRP in the early years (49). Therefore, in the mediation study, we explored the mediating effect of CRP and BMR on the causality between glucosamine and indicators of cognition and sarcopenia. The findings indicated that CRP and BMR might potentially act as mediators in the causative relationship between glucosamine and sarcopenia proxy indicators. Additionally, BMR could potentially act as a mediator in the causal relationship between glucosamine and cognitive proxy indicators. It is crucial to acknowledge the significance of using glucosamine, regulating CRP levels, and improving and resolving the drop in BMR to address the gradual aging of the population, including cognitive loss and the development of sarcopenia.

The mechanism of action of glucosamine has been systematically studied *in vitro* and *in vivo* animal experiments. Weimer et al. found that glucosamine prolonged the lifespan of Caenorhabditis elegans and C57BL/6 mice, which may be related to the mimicking of a low-carbohydrate diet, the activation of AMP-activated protein kinase (AMPK/AAK-2), and an increase in mitochondrial biosynthesis (50). Besides, Chou et al. discovered that daily intraperitoneal injections of glucosamine (0, 3, 10, and 30 mg/each) in 7-week-old C57BL/6 mice significantly improved cognitive function in a novel object recognition experiment, which could be related to the fact that glucosamine increases BDNF levels by interfering with the cAMP/PKA/CREB-dependent pathway in the hippocampus (51). Chao et al. investigated the effects of glucosamine on mice fed either a normal diet or a high-fat diet, and the effects of glucosamine on mice (7-week-old male C57BL/6 mice were given glucosamine (0, 3, 10, or 30 mg/each/day) for 14 consecutive days over a 10-week period), HT22 hippocampal cells, STHdhQ7/Q7 striatal cells, and rat primary cortical neurons were affected. Glucosamine may enhance learning and memory in mice by activating NF-κB, Akt, p38, and PKA/CREB pathways. This, in turn, promotes FGF21 production in the brain (52) Lee et al. found that glucosamine pretreatment inhibits hypoxia-induced neuroinflammation and learning memory deficits in adult zebrafish. This may be associated with glucosamine intervention in the hexosamine metabolic pathway (increasing levels of neurotransmitters γ-aminobutyric acid, glutamate, and acetylcholine) (53).

Although our research has a substantial sample size, it is susceptible to many constraints. Primarily, the data for all participants in this research mostly originated from European nations, thereby limiting the generalizability of the findings to other ethnic contexts. Secondly, because of the use of summary-level statistics in the published data, we were unable to examine a non-linear causal relationship between glucosamine and indicators of cognition and sarcopenia. Subsequent investigations should prioritize this issue. Furthermore, it is important to acknowledge that MR analysis provides less compelling causality evidence than RCTs. Therefore, additional high-quality RCT evidence is still required to complement and reinforce our findings.

In conclusion, the present study identified glucosamine use as a protective factor for enhanced cognition (cognitive performance and FIS increased) and decreased aging and sarcopenia (ALM, WBFM, usual walking pace and physical activity increased, FA, and DNA methylation GrimAge acceleration decreased). The findings also suggest the need to improve and treat elevated CRP levels and reduced BMR to prevent cognitive decline and accelerated aging, as well as the development of sarcopenia risk.

## Data Availability

The original contributions presented in the study are included in the article/**Supplementary Material**. Further inquiries can be directed to the corresponding authors.
