# Supplementary material for: Causality of genetically determined glucosamine supplementation on cognition and sarcopenia: a Mendelian randomization study
Source: Front Endocrinol (Lausanne). 2024 Dec 23;15:1404308. doi: 10.3389/fendo.2024.1404308 (PMC11700805; doi:10.3389/fendo.2024.1404308)
Supplement: Supplementary file 1 [file DataSheet1.docx]

**STROBE-MR checklist of recommended items to address in reports of Mendelian randomization studies**^1^ ^2^

| **Item No.** | **Section** | **Checklist item** | **Page No.** | **Relevant text from manuscript** |
| --- | --- | --- | --- | --- |
| 1 | **TITLE and ABSTRACT** | Indicate Mendelian randomization (MR) as the study’s design in the title and/or the abstract if that is a main purpose of the study | 1-2 in title and abstract | **Title:** Genetic contribution to the comorbidity  for the effect of glucosamine  supplementation on cognition and  sarcopenia: a Mendelian randomization  study  **Background in the abstract:** This study aims to verify whether glucosamine is causally associated with cognitive function and sarcopenia. |
|  | **INTRODUCTION** |  |  |  |
| 2 | **Background** | Explain the scientific background and rationale for the reported study. What is the exposure? Is a potential causal relationship between exposure and outcome plausible? Justify why MR is a helpful method to address the study question | 2-3 in Introduction | **Scientific background and rationale:** The phenomenon of aging societies is becoming a prevalent worldwide trend. Epidemiological studies indicate that now, 11% of the global population is aged 60 or over. Projections suggest that by the year 2050, this percentage will double to reach 22% of the population. Notably, the prevalence of aging populations has resulted in the growth of certain chronic age-related ailments, such as cognitive decline, neurodegenerative changes, and sarcopenia, even among older adults who do not develop dementia. Due to the lack of effective pharmacological interventions for these social and public health issues, there has been a significant focus on thoroughly investigating potentially modifiable protective factors.  **What is the exposure? Is a potential causal relationship between exposure and outcome plausible?**  Glucosamine, classified as an amino sugar, is a naturally derived compound that promotes the synthesis of glycosaminoglycans. several studies suggest that regular glucosamine supplementation may confer a protective effect against mortality in the general population and mitigate age-related cognitive decline, potentially extending their overall health span. Moreover, study data has pointed to increased life expectancy and decreased incident vascular dementia in individuals who are supplemented with glucosamine.  **Justify why MR is a helpful method to address the study question**  Due to limitations such as residual confounding, potential reverse causality, inadequate adjustment, and a focus on correlation rather than causation, observational studies have been hindered in identifying a causal effect of glucosamine supplementation on cognition and sarcopenia. On the other hand, the implementation of large-scale randomized controlled trials (RCTs) poses challenges. Utilizing genetic variants as proxies for glucosamine, Mendelian randomization (MR) circumvents several limitations and offers genetic evidence supporting causal associations. |
| 3 | **Objectives** | State specific objectives clearly, including pre-specified causal hypotheses (if any). State that MR is a method that, under specific assumptions, intends to estimate causal effects | 2-3 in Introduction | Utilizing genetic variants as proxies for glucosamine, mendelian randomization (MR) circumvents several limitations and offers genetic evidence supporting causal associations. We performed the present MR study with the aim of evaluating the causality between glucosamine and changes in aging and cognition by analysing the summary-level genome-wide association studies (GWAS) data of glucosamine, cognition proxy indicators as well as aging and sarcopenia-related indicators such as cognitive performance, facial aging, appendicular lean mass (ALM), whole body fat-free mass (WBFM), grip strength, DNA methylation GrimAge acceleration, usual walking pace and other relevant traits. Therefore, we hypothesize that regular glucosamine use may causally impact cognition and sarcopenia-related traits. |
|  | **METHODS** |  |  |  |
| 4 | **Study design and data sources** | Present key elements of the study design early in the article. Consider including a table listing sources of data for all phases of the study. For each data source contributing to the analysis, describe the following: | 3-5 and Figure 1 |  |
|  | a) | Setting: Describe the study design and the underlying population, if possible. Describe the setting, locations, and relevant dates, including periods of recruitment, exposure, follow-up, and data collection, when available. | 3-5 in Method part and Supplementary Table 1. | A two-sample MR was utilized to assess the causal association of glucosamine on cognition and sarcopenia-related traits. Briefly, glucosamine served as the exposures, while two indicators of cognition, and seven sarcopenia‐related traits served as the outcomes. This two-sample MR study consisted of two phases of analysis. In the first phase, we first examined the causal effects of glucosamine on 2 cognitive traits and 7 sarcopenia-related traits and then investigated whether these causal effects were independent of OA and BMI. In the second phase, we assessed the mediating roles of CRP and BMR in the causal associations between glucosamine and the outcomes of cognitive function and sarcopenia-related traits. MR was performed under three crucial assumptions. (1) genetic IVs are associated with glucosamine (the relevance assumption); (2) genetic IVs are independent of confounding variables (the independence assumption); (3) genetic IVs only affect outcomes above through glucosamine (the exclusion restriction assumption)  Genetic variants significantly associated with glucosamine were extracted from a large Open GWAS data of the Medical Research Council Integrative Epidemiology Unit (MRC-IEU) consortium, which comprised 89,339 cases and 372,045 controls, available through the UK Biobank (https://gwas.mrcieu.ac.uk/datasets/ukb-b-11535/). A baseline touchscreen questionnaire was used to acquire habitual glucosamine information. The participants were asked, "Do you regularly take any of the following?" The touchscreen questionnaire allowed participants to pick various answers from a variety of supplemental materials (Mineral and other dietary supplements). We classified glucosamine users as follows: 0 no; 1 yes.  Cognition outcomes included cognitive performance and fluid intelligence score (FIS) traits. cognitive performance data were extracted from the largest publicly available GWAS in 257,841 European-ancestry participants from COGENT Consortium (35 sub-cohorts) and UK Biobank, with adjustment for sex, age, and population stratification. GWAS data for FIS data were from the Medical Research Council Integrative Epidemiology Unit (MRC-IEU) consortium, available through the UK Biobank. Seven traits were selected for valid predictors of sarcopenia: appendicular lean mass (ALM), whole body fat-free mass (WBFM), low hand grip strength, facial aging (FA), moderate to vigorous physical activity levels, usual walking pace and DNA methylation GrimAge acceleration. Among them, grip strength was used to measure muscle strength, and WBFM and ALM were used to measure muscle mass. ALM is predominantly determined by skeletal muscle and has been shown to make up ≥75% of skeletal muscle in the body, whereas WBFM comprises smooth muscle, cardiac muscle, and skeletal muscle. The GWAS meta-analysis of 256,523 Europeans aged 60 years and older from 22 cohorts was utilized to extract grip strength data; the criteria for this analysis were established by the European Working Group on Sarcopenia in Older People (EWGSOP) consortium (grip strength <30 kg Male; <20 kg Female).  FA serves as a prominent visible indicator of aging. The summary statistics for facial aging of GWAS were obtained from the UK Biobank, which contained a total of 423,999 participants of European ancestry (194,391 males and 229,601 females, aged 40–69). Facial ageing (FA) is evaluated with non-subjective perceived age through a touchscreen questionnaire. 103,300 individuals reported appearing to be their age. A total of 312,062 individuals reported appearing younger than their biological age, while 8,630 individuals reported appearing older than their biological age. The participants were asked “Do people say that you look” and allowed to pick the answer from a variety of supplemental materials. The participants were coded by the researchers in accordance with their actual age and perceived age. Participants were assigned codes as follows: 0 indicated that they appeared older, 1 indicated that they appeared younger, 0.5 indicated that they appeared to be their age (third-party observations, including those of non-participants and non-researchers, were conducted without knowledge of the participants' actual ages). The summary statistics for moderate to vigorous physical activity levels were derived from a GWAS conducted in 377,234 UK Biobank participants. Just as low physical performance is one of the characteristics of sarcopenia, clinical walking speed is a fast, safe and highly reliable test for sarcopenia. Usual walking pace data were obtained from the UK Biobank with 459,915 European individuals. DNA GrimAge acceleration is a measure of accelerated biological aging. It is established by combining chronological age, sex, and surrogate biomarkers based on DNA methylation for seven plasma proteins and smoking pack-years. The data for DNA methylation GrimAge acceleration was derived from McCartney et al. GWAS study with 34,467 subjects.  Oral glucosamine has traditionally been advised for the management of knee and hip OA. Therefore, OA was selected and identified as a major confounder. Summary statistics for OA were obtained from the largest publicly available GWAS with 484,598 participants (39,515 OA cases and 445,083 controls), which utilized the UK Biobank data. Besides, we also assessed the effect of body mass index (BMI) as a confounder. BMI data were derived from the largest publicly available GWAS by the Genetic Investigation of Anthropometric Traits (GIANT) consortium including 681,275 European individuals. C-reactive protein (CRP) levels may be involved in altered cognitive function and accelerated aging. Besides, the development of sarcopenia usually brings about a decrease in the basic metabolic rate (BMR) of the organism, a decrease in trunk activity. Therefore, CRP and BMR were selected and identified as the mediators in the effect of glucosamine on cognition and sarcopenia‐related traits. CRP data were obtained from the largest publicly available GWAS with 575,531 participants of European ancestry, which conducted in the UK Biobank participants (427,367 European descent) and the Cohorts for Heart and Aging Research in Genomic Epidemiology (CHARGE) Consortium (575,531 European descent). Summary statistics for basal metabolic rate were derived from MRC-IEU consortium, available through the UK Biobank, which included 454,874 European participants |
|  | b) | Participants: Give the eligibility criteria, and the sources and methods of selection of participants. Report the sample size, and whether any power or sample size calculations were carried out prior to the main analysis | 3-5 in manuscript, and Supplementary Table 1. | Summary statistics were obtained from the reputable publicly available GWAS conducted in European participants;  Genetic variants significantly associated with glucosamine were extracted from a large Open GWAS data of the Medical Research Council Integrative Epidemiology Unit (MRC-IEU) consortium, which comprised 89,339 cases and 372,045 controls, available through the UK Biobank (https://gwas.mrcieu.ac.uk/datasets/ukb-b-11535/). A baseline touchscreen questionnaire was used to acquire habitual glucosamine information. The participants were asked, "Do you regularly take any of the following?" The touchscreen questionnaire allowed participants to pick various answers from a variety of supplemental materials (Mineral and other dietary supplements). We classified glucosamine users as follows: 0 no; 1 yes.  Cognition outcomes included cognitive performance and fluid intelligence score (FIS) traits. cognitive performance data were extracted from the largest publicly available GWAS in 257,841 European-ancestry participants from COGENT Consortium (35 sub-cohorts) and UK Biobank, with adjustment for sex, age, and population stratification. GWAS data for FIS data were from the Medical Research Council Integrative Epidemiology Unit (MRC-IEU) consortium, available through the UK Biobank. Seven traits were selected for valid predictors of sarcopenia: appendicular lean mass (ALM), whole body fat-free mass (WBFM), low hand grip strength, facial aging (FA), moderate to vigorous physical activity levels, usual walking pace and DNA methylation GrimAge acceleration. Among them, grip strength was used to measure muscle strength, and WBFM and ALM were used to measure muscle mass. ALM is predominantly determined by skeletal muscle and has been shown to make up ≥75% of skeletal muscle in the body, whereas WBFM comprises smooth muscle, cardiac muscle, and skeletal muscle. The GWAS meta-analysis of 256,523 Europeans aged 60 years and older from 22 cohorts was utilized to extract grip strength data; the criteria for this analysis were established by the European Working Group on Sarcopenia in Older People (EWGSOP) consortium (grip strength <30 kg Male; <20 kg Female).  FA serves as a prominent visible indicator of aging. The summary statistics for facial aging of GWAS were obtained from the UK Biobank, which contained a total of 423,999 participants of European ancestry (194,391 males and 229,601 females, aged 40–69). Facial ageing (FA) is evaluated with non-subjective perceived age through a touchscreen questionnaire. 103,300 individuals reported appearing to be their age. A total of 312,062 individuals reported appearing younger than their biological age, while 8,630 individuals reported appearing older than their biological age. The participants were asked “Do people say that you look” and allowed to pick the answer from a variety of supplemental materials. The participants were coded by the researchers in accordance with their actual age and perceived age. Participants were assigned codes as follows: 0 indicated that they appeared older, 1 indicated that they appeared younger, 0.5 indicated that they appeared to be their age (third-party observations, including those of non-participants and non-researchers, were conducted without knowledge of the participants' actual ages). The summary statistics for moderate to vigorous physical activity levels were derived from a GWAS conducted in 377,234 UK Biobank participants. Just as low physical performance is one of the characteristics of sarcopenia, clinical walking speed is a fast, safe and highly reliable test for sarcopenia. Usual walking pace data were obtained from the UK Biobank with 459,915 European individuals. DNA GrimAge acceleration is a measure of accelerated biological aging. It is established by combining chronological age, sex, and surrogate biomarkers based on DNA methylation for seven plasma proteins and smoking pack-years. The data for DNA methylation GrimAge acceleration was derived from McCartney et al. GWAS study with 34,467 subjects.  Oral glucosamine has traditionally been advised for the management of knee and hip OA. Therefore, OA was selected and identified as a major confounder. Summary statistics for OA were obtained from the largest publicly available GWAS with 484,598 participants (39,515 OA cases and 445,083 controls), which utilized the UK Biobank data. Besides, we also assessed the effect of body mass index (BMI) as a confounder. BMI data were derived from the largest publicly available GWAS by the Genetic Investigation of Anthropometric Traits (GIANT) consortium including 681,275 European individuals. C-reactive protein (CRP) levels may be involved in altered cognitive function and accelerated aging. Besides, the development of sarcopenia usually brings about a decrease in the basic metabolic rate (BMR) of the organism, a decrease in trunk activity. Therefore, CRP and BMR were selected and identified as the mediators in the effect of glucosamine on cognition and sarcopenia‐related traits. CRP data were obtained from the largest publicly available GWAS with 575,531 participants of European ancestry, which conducted in the UK Biobank participants (427,367 European descent) and the Cohorts for Heart and Aging Research in Genomic Epidemiology (CHARGE) Consortium (575,531 European descent). Summary statistics for basal metabolic rate were derived from MRC-IEU consortium, available through the UK Biobank, which included 454,874 European participants |
|  | c) | Describe measurement, quality control and selection of genetic variants | 3-6 | Genetic variants associated with glucosamine at genome-wide significance (p < 5×10^-6^) were identified and extracted as potential IVs. Then, we performed the clumping procedure (linkage disequilibrium r^2^ < 0.001 within kb = 10,000) to ensure that genetic variants were independent of each other. To assess weak instrumental bias, the F statistics were calculated using the following formula: F = R^2^ (N–k–1)/[(1–R^2^) k], where N represents the sample size and k denotes the number of included SNPs, R^2^ represents the proportion of variability that can be attributed to each SNP. Besides, R^2^ can be calculated by the formula: R^2^ = 2 × β^2^ × EAF × (1 – EAF), where βindicated the estimated effect size of the IVs and EAF represents effect allele frequency. A genetic variation used as a weak IVs is considered when the F-statistic is less than 10, as it can potentially introduce bias to the results. Details of GWAS studies are provided in the Supplementary Table 1. |
|  | d) | For each exposure, outcome, and other relevant variables, describe methods of assessment and diagnostic criteria for diseases | 3-5 | Genetic variants significantly associated with glucosamine were extracted from a large Open GWAS data of the Medical Research Council Integrative Epidemiology Unit (MRC-IEU) consortium, which comprised 89,339 cases and 372,045 controls, available through the UK Biobank (https://gwas.mrcieu.ac.uk/datasets/ukb-b-11535/). A baseline touchscreen questionnaire was used to acquire habitual glucosamine information. The participants were asked, "Do you regularly take any of the following?" The touchscreen questionnaire allowed participants to pick various answers from a variety of supplemental materials (Mineral and other dietary supplements). We classified glucosamine users as follows: 0 no; 1 yes.  Cognition outcomes included cognitive performance and fluid intelligence score (FIS) traits. cognitive performance data were extracted from the largest publicly available GWAS in 257,841 European-ancestry participants from COGENT Consortium (35 sub-cohorts) and UK Biobank, with adjustment for sex, age, and population stratification. GWAS data for FIS data were from the Medical Research Council Integrative Epidemiology Unit (MRC-IEU) consortium, available through the UK Biobank. Seven traits were selected for valid predictors of sarcopenia: appendicular lean mass (ALM), whole body fat-free mass (WBFM), low hand grip strength, facial aging (FA), moderate to vigorous physical activity levels, usual walking pace and DNA methylation GrimAge acceleration. Among them, grip strength was used to measure muscle strength, and WBFM and ALM were used to measure muscle mass. ALM is predominantly determined by skeletal muscle and has been shown to make up ≥75% of skeletal muscle in the body, whereas WBFM comprises smooth muscle, cardiac muscle, and skeletal muscle. The GWAS meta-analysis of 256,523 Europeans aged 60 years and older from 22 cohorts was utilized to extract grip strength data; the criteria for this analysis were established by the European Working Group on Sarcopenia in Older People (EWGSOP) consortium (grip strength <30 kg Male; <20 kg Female).  FA serves as a prominent visible indicator of aging. The summary statistics for facial aging of GWAS were obtained from the UK Biobank, which contained a total of 423,999 participants of European ancestry (194,391 males and 229,601 females, aged 40–69). Facial ageing (FA) is evaluated with non-subjective perceived age through a touchscreen questionnaire. 103,300 individuals reported appearing to be their age. A total of 312,062 individuals reported appearing younger than their biological age, while 8,630 individuals reported appearing older than their biological age. The participants were asked “Do people say that you look” and allowed to pick the answer from a variety of supplemental materials. The participants were coded by the researchers in accordance with their actual age and perceived age. Participants were assigned codes as follows: 0 indicated that they appeared older, 1 indicated that they appeared younger, 0.5 indicated that they appeared to be their age (third-party observations, including those of non-participants and non-researchers, were conducted without knowledge of the participants' actual ages). The summary statistics for moderate to vigorous physical activity levels were derived from a GWAS conducted in 377,234 UK Biobank participants. Just as low physical performance is one of the characteristics of sarcopenia, clinical walking speed is a fast, safe and highly reliable test for sarcopenia. Usual walking pace data were obtained from the UK Biobank with 459,915 European individuals. DNA GrimAge acceleration is a measure of accelerated biological aging. It is established by combining chronological age, sex, and surrogate biomarkers based on DNA methylation for seven plasma proteins and smoking pack-years. The data for DNA methylation GrimAge acceleration was derived from McCartney et al. GWAS study with 34,467 subjects.  Oral glucosamine has traditionally been advised for the management of knee and hip OA. Therefore, OA was selected and identified as a major confounder. Summary statistics for OA were obtained from the largest publicly available GWAS with 484,598 participants (39,515 OA cases and 445,083 controls), which utilized the UK Biobank data. Besides, we also assessed the effect of body mass index (BMI) as a confounder. BMI data were derived from the largest publicly available GWAS by the Genetic Investigation of Anthropometric Traits (GIANT) consortium including 681,275 European individuals. C-reactive protein (CRP) levels may be involved in altered cognitive function and accelerated aging. Besides, the development of sarcopenia usually brings about a decrease in the basic metabolic rate (BMR) of the organism, a decrease in trunk activity. Therefore, CRP and BMR were selected and identified as the mediators in the effect of glucosamine on cognition and sarcopenia‐related traits. CRP data were obtained from the largest publicly available GWAS with 575,531 participants of European ancestry, which conducted in the UK Biobank participants (427,367 European descent) and the Cohorts for Heart and Aging Research in Genomic Epidemiology (CHARGE) Consortium (575,531 European descent). Summary statistics for basal metabolic rate were derived from MRC-IEU consortium, available through the UK Biobank, which included 454,874 European participants |
|  | e) | Provide details of ethics committee approval and participant informed consent, if relevant | 3 | The included researches and databases had obtained the ethical committee approval and informed consent from participants. |
| 5 | **Assumptions** | Explicitly state the three core IV assumptions for the main analysis (relevance, independence and exclusion restriction) as well assumptions for any additional or sensitivity analysis | 3, and Figure 1 | MR was performed under three crucial assumptions. (1) genetic IVs are associated with glucosamine (the relevance assumption); (2) genetic IVs are independent of confounding variables (the independence assumption); (3) genetic IVs only affect outcomes above through glucosamine (the exclusion restriction assumption).  MVMR provides insights into the extent to which the mediator explains the relationship between the exposure and the outcome. In addition, MVMR allows for the examination of exposure simultaneously while considering potential confounding factors. The IVW method was used for the MVMR. Moreover, we performed two‐step MR to explore whether CRP levels and BMR has mediation effect on the causal associations between glucosamine and the cognition and sarcopenia‐related outcomes. The first step was to use UVMR to estimate the causal effect of glucosamine on mediators (CRP and BMR, separately), with each estimate represented as β1. The second step was to estimate the causal effect of the mediators on outcomes (cognition and sarcopenia‐related traits), with adjustment for glucosamine using MVMR (MVMR estimate represented as β2). The entire effect of glucosamine on outcomes can be split into direct (not mediated by mediators) and indirect effects (effect mediated by the mediators). The following conditions exist: 1) If the coefficients β0, β1, and β2 are all statistically significant, it suggests that there is a causal relationship between the exposure and outcome variables. Furthermore, this relationship may be influenced to some extent by mediating factors. The mediation proportion of mediator (CRP and BMR) in the causal association between glucosamine and outcomes was calculated as the product of β1 and β2 divided by β0; 2) If the coefficient β0 is not statistically significant, yet the coefficients β1 and β2 are both statistically significant, it suggests that the impact is mostly influenced by the mediator variable; 3) If the coefficient β0 is statistically significant and either β1 or β2 is not statistically significant, then indicates that the mediating effect does not play a role in mediating the relationship between exposure and the result. |
| 6 | **Statistical methods: main analysis** | Describe statistical methods and statistics used | 5-6 |  |
|  | a) | Describe how quantitative variables were handled in the analyses (i.e., scale, units, model) | Not applicable | Not applicable |
|  | b) | Describe how genetic variants were handled in the analyses and, if applicable, how their weights were selected | 5-6 | Genetic variants associated with glucosamine at genome-wide significance (p < 5×10^-6^) were identified and extracted as potential IVs. Then, we performed the clumping procedure (linkage disequilibrium r^2^ < 0.001 within kb = 10,000) to ensure that genetic variants were independent of each other.  Furthermore, the confounding analysis was performed utilizing Phenoscanner V2 (http://www.phenoscanner.medschl.cam.ac.uk/). We have identified certain diseases or physical ailments that have a high correlation with IVs at a significance level of p < 5 × 10^-6^. Subsequently, we condensed and examined pertinent data pertaining to IVS, GWASes, and illnesses. This approach not only assists in identifying important factors that need to be accounted for in MVMR, but it also enables us to explore the process of mediation and potential causal pathways. |
|  | c) | Describe the MR estimator (e.g. two-stage least squares, Wald ratio) and related statistics. Detail the included covariates and, in case of two-sample MR, whether the same covariate set was used for adjustment in the two samples | 5-6 | MR estimates were reported as odds ratios (ORs) with 95% confidence intervals (CIs). We evaluated the causal relationship of glucosamine with two cognition traits and seven sarcopenia-related traits by applying univariable MR analysis (UVMR), with estimates represented as β0. Then we applied multivariable MR (MVMR) to evaluate the causal effect of glucosamine on cognition and sarcopenia-related traits with both adjustment for OA and BMI to determine whether glucosamine was causally associated with the cognition and sarcopenia-related outcomes independent of OA and BMI.  We applied fixed effects inverse variance weighted (IVW-FE) as the major analysis for UVMR. To enhance the robustness of IVW-FE results, we also employed MR Egger, maximum likelihood, weighted median, and weighted mode methods for additional validation. The MR-Egger method aids in the evaluation of horizontal pleiotropy and bias caused by weak instruments. Weighted median approach could effectively mitigate the impact of outlier SNPs on the results, thereby enhancing robustness. The weighted mode approach provides dependable estimates when the majority of similar individual-instrument causal effect estimates are derived from genuine instrumental variables, even if a large portion of them is invalid. Sensitivity analyses were conducted to assess potential biases and underlying assumptions in the MR analysis, aiming to validate and refine the results. Heterogeneity was assessed using the Cochran Q test. If the p-value of Cochran's Q test is greater than 0.05, there is no heterogeneity. Once heterogeneity (*p* < 0.05) has been identified, use the multiplicative random effects IVW (IVW-MRE) method to ascertain the causal influence. Besides, the MR pleiotropy residual sum and outlier (MR-PRESSO) test and MR-Egger were performed to assess possible horizontal pleiotropy. Horizontal pleiotropy is described as some instruments’ additional biological effects that influence the outcome independently of the exposure. The presence of horizontal pleiotropy can be detected using the MR-PRESSO global test, while the MR-PRESSO outlier test is utilized to exclude outlier SNPs and assesses the impact of excluding these SNPs on causal estimations. The MR-Egger intercept test is additionally employed to assess the horizontal pleiotropy. A leave-one-out method was employed to identify high-influence points as whether the significant results were influenced by a particular SNP. |
|  | d) | Explain how missing data were addressed | Not applicable | Not applicable |
|  | e) | If applicable, indicate how multiple testing was addressed | 6 | To adjust for multiple testing (several exposures), the MR effect estimates were statistically significant at less than five percent using the Benjamini-Hochberg false discovery rate (FDR). The results were exhibited in Supplementary Table 8 and 9. |
| 7 | **Assessment of assumptions** | Describe any methods or prior knowledge used to assess the assumptions or justify their validity | 5-6 | We applied fixed effects inverse variance weighted (IVW-FE) as the major analysis for UVMR. To enhance the robustness of IVW-FE results, we also employed MR Egger, maximum likelihood, weighted median, and weighted mode methods for additional validation. The MR-Egger method aids in the evaluation of horizontal pleiotropy and bias caused by weak instruments. Weighted median approach could effectively mitigate the impact of outlier SNPs on the results, thereby enhancing robustness. The weighted mode approach provides dependable estimates when the majority of similar individual-instrument causal effect estimates are derived from genuine instrumental variables, even if a large portion of them is invalid. Sensitivity analyses were conducted to assess potential biases and underlying assumptions in the MR analysis, aiming to validate and refine the results. Heterogeneity was assessed using the Cochran Q test. If the p-value of Cochran's Q test is greater than 0.05, there is no heterogeneity. Once heterogeneity (*p* < 0.05) has been identified, use the multiplicative random effects IVW (IVW-MRE) method to ascertain the causal influence. Besides, the MR pleiotropy residual sum and outlier (MR-PRESSO) test and MR-Egger were performed to assess possible horizontal pleiotropy. Horizontal pleiotropy is described as some instruments’ additional biological effects that influence the outcome independently of the exposure. The presence of horizontal pleiotropy can be detected using the MR-PRESSO global test, while the MR-PRESSO outlier test is utilized to exclude outlier SNPs and assesses the impact of excluding these SNPs on causal estimations (34). The MR-Egger intercept test is additionally employed to assess the horizontal pleiotropy. leave-one-out method was employed to identify high-influence points as whether the significant results were influenced by a particular SNP. |
| 8 | **Sensitivity analyses and additional analyses** | Describe any sensitivity analyses or additional analyses performed (e.g. comparison of effect estimates from different approaches, independent replication, bias analytic techniques, validation of instruments, simulations) | 5-6 | We applied fixed effects inverse variance weighted (IVW-FE) as the major analysis for UVMR. To enhance the robustness of IVW-FE results, we also employed MR Egger, maximum likelihood, weighted median, and weighted mode methods for additional validation. The MR-Egger method aids in the evaluation of horizontal pleiotropy and bias caused by weak instruments. Weighted median approach could effectively mitigate the impact of outlier SNPs on the results, thereby enhancing robustness. The weighted mode approach provides dependable estimates when the majority of similar individual-instrument causal effect estimates are derived from genuine instrumental variables, even if a large portion of them is invalid Sensitivity analyses were conducted to assess potential biases and underlying assumptions in the MR analysis, aiming to validate and refine the results. Heterogeneity was assessed using the Cochran Q test. If the p-value of Cochran's Q test is greater than 0.05, there is no heterogeneity. Once heterogeneity (*p* < 0.05) has been identified, use the multiplicative random effects IVW (IVW-MRE) method to ascertain the causal influence. Besides, the MR pleiotropy residual sum and outlier (MR-PRESSO) test and MR-Egger were performed to assess possible horizontal pleiotropy. Horizontal pleiotropy is described as some instruments’ additional biological effects that influence the outcome independently of the exposure. The presence of horizontal pleiotropy can be detected using the MR-PRESSO global test, while the MR-PRESSO outlier test is utilized to exclude outlier SNPs and assesses the impact of excluding these SNPs on causal estimations. The MR-Egger intercept test is additionally employed to assess the horizontal pleiotropy. A leave-one-out method was employed to identify high-influence points as whether the significant results were influenced by a particular SNP. |
| 9 | **Software and pre-registration** |  |  |  |
|  | a) | Name statistical software and package(s), including version and settings used | 5-6 | A significance level of 0.05 was implemented for the p-value. To adjust for multiple testing (several exposures), the MR effect estimates were statistically significant at less than five percent using the Benjamini-Hochberg false discovery rate (FDR). All analyses were carried out using packages “TwoSampleMR”, “MendelianRandomization”, “MR-PRESSO”, and “Phenoscanner” in R version 4.3.1. |
|  | b) | State whether the study protocol and details were pre-registered (as well as when and where) | Not applicable | Not applicable. The study protocol and details have not been pre-registered. |
|  | **RESULTS** |  |  |  |
| 10 | **Descriptive data** |  |  |  |
|  | a) | Report the numbers of individuals at each stage of included studies and reasons for exclusion. Consider use of a flow diagram | Not applicable | Not applicable |
|  | b) | Report summary statistics for phenotypic exposure(s), outcome(s), and other relevant variables (e.g. means, SDs, proportions) | 7 in Result part and Figure 3 | After analysing and evaluating the related information about glucosamine associated SNPs, GWASes and Diseases through Phenoscanner, we found some potential confounders or outcomes, mainly including arm fat-free mass, arm predicted mass, chronotype, morning or evening person, forced expiratory volume in 1-second, forced vital capacity, and qualifications, etc. |
|  | c) | If the data sources include meta-analyses of previous studies, provide the assessments of heterogeneity across these studies | 6-8 in Result part and Supplementary Table 3, 4 and 5 | Besides, due to the detected some heterogeneity by Cochran Q statistic (*p* < 0.01), we applied the IVW-MRE method and also detected consistent results (Supplementary Table 3, 4 and 5). |
|  | d) | For two-sample MR:  i.  Provide justification of the similarity of the genetic variant-exposure associations between the exposure and outcome samples  ii.  Provide information on the number of individuals who overlap between the exposure and outcome studies | 6-8 | Confounding analysis  After analyzing and evaluating the related information about glucosamine associated SNPs, GWASes and Diseases through Phenoscanner, we found some potential confounders or outcomes, mainly including arm fat-free mass, arm predicted mass, chronotype, morning or evening person, forced expiratory volume in 1-second, forced vital capacity, and qualifications, etc. (Figure 3). |
| 11 | **Main results** |  |  |  |
|  | a) | Report the associations between genetic variant and exposure, and between genetic variant and outcome, preferably on an interpretable scale | 6-8 | The main results of MR analysis are shown in Figure 2. We performed an UVMR analysis to investigate the association between glucosamine and cognition traits. The main analytical method applied in the MR analysis, IVW-FE discovered that taking glucosamine significantly increased cognitive performance (OR = 1.40, 95% CI = 1.15-1.70, *p* = 8.46E-04), and FIS (OR = 2.50, CI = 1.47-4.26, *p* = 7.50E-04) (Figure 2, Supplementary Table 3 and 4).  The IVW-FE method showed that glucosamine was associated with increased ALM (OR = 1.44, 95% CI = 1.26-1.65, *p* = 6.45E-08), WBFM (OR = 1.15, 95% CI = 1.05-1.25, *p* = 1.97E-03), usual walking pace (OR = 1.26, 95% CI = 1.16-1.38, *p* = 2.55E-07) as well as moderate to vigorous physical activity levels (OR = 1.26, 95% CI = 1.08-1.47, *p* = 3.29E-03), but associated with decreased FA risk (OR = 0.85, 95% CI = 0.79-0.92, *p* = 3.77E-05) and decreased DNA methylation GrimAge acceleration (OR = 0.02, 95% CI = 0.002-0.21, *p* = 0.001) (Figure 2, Supplementary Table 3 and 4). Besides, there was no significant causal associations between glucosamine and low hand grip strength (OR = 1.17, 95% CI = 0.69-1.97, *p* = 0.56). |
|  | b) | Report MR estimates of the relationship between exposure and outcome, and the measures of uncertainty from the MR analysis, on an interpretable scale, such as odds ratio or relative risk per SD difference | 6-8 | The main results of MR analysis are shown in Figure 2. We performed an UVMR analysis to investigate the association between glucosamine and cognition traits. The main analytical method applied in the MR analysis, IVW-FE discovered that taking glucosamine significantly increased cognitive performance (OR = 1.40, 95% CI = 1.15-1.70, *p* = 8.46E-04), and FIS (OR = 2.50, CI = 1.47-4.26, *p* = 7.50E-04) (Figure 2, Supplementary Table 3 and 4).  The IVW-FE method showed that glucosamine was associated with increased ALM (OR = 1.44, 95% CI = 1.26-1.65, *p* = 6.45E-08), WBFM (OR = 1.15, 95% CI = 1.05-1.25, *p* = 1.97E-03), usual walking pace (OR = 1.26, 95% CI = 1.16-1.38, *p* = 2.55E-07) as well as moderate to vigorous physical activity levels (OR = 1.26, 95% CI = 1.08-1.47, *p* = 3.29E-03), but associated with decreased FA risk (OR = 0.85, 95% CI = 0.79-0.92, *p* = 3.77E-05) and decreased DNA methylation GrimAge acceleration (OR = 0.02, 95% CI = 0.002-0.21, *p* = 0.001) (Figure 2, Supplementary Table 3 and 4). Besides, there was no significant causal associations between glucosamine and low hand grip strength (OR = 1.17, 95% CI = 0.69-1.97, *p* = 0.56).  We performed MVMR Analysis by both adjusted OA and BMI to further assess the associations between glucosamine and outcomes. After adjusting both OA and BMI, we found that taking glucosamine continued to have a significant effect on cognitive performance (OR = 1.87, 95% CI = 1.23-2.83, *p* = 3.42E-03), FIS (OR = 4.88, CI = 1.87-12.73, *p* = 1.18E-03), ALM (OR = 3.59, 95% CI = 2.29-5.63, *p* = 2.66E-08), WBFM (OR = 1.66, 95% CI = 1.33-2.08, *p* = 7.38E-06), usual walking pace (OR = 1.53, 95% CI = 1.34-1.76, *p* = 9.46E-10), and moderate to vigorous physical activity levels (OR = 1.40, 95% CI = 1.12-1.74, *p* = 3.25E-03) (Supplementary Table 8).  We further explored the mediating roles of CRP and BMR, in the improvement of cognition and sarcopenia‐related traits by glucosamine using MVMR coupled with a two-step MR method, respectively (Supplementary Table 9). The protective effects of glucosamine on ALM, WBFM, usual walking pace, and moderate to vigorous physical activity levels were continued after adjusting for CRP. However, the effects of glucosamine on cognitive performance, FIS, FA, DNA methylation GrimAge acceleration and low hand grip strength were weakening after adjusting for CRP, suggesting this association was largely affected by CRP. The proportion mediated by CRP in the associations between glucosamine and outcomes was 4% for ALM, 1.4% for WBFM, 5% for usual walking pace, and 3% for moderate to vigorous physical activity levels (Table 1).  Moreover, the protective effects of glucosamine on cognitive performance, ALM, WBFM, usual walking pace, and moderate to vigorous physical activity levels were continued after adjusting for BMR. However, the effects of glucosamine on FIS, FA, and DNA methylation GrimAge acceleration were weakening after adjusting for BMR, suggesting this association was largely affected by BMR. Specifically, the proportion mediated by BMR in the associations between glucosamine and outcomes was and 3% for cognitive performance, 3% for FIS, 39% for ALM, 71% for WBFM, and 3% for usual walking pace (Table 2). |
|  | c) | If relevant, consider translating estimates of relative risk into absolute risk for a meaningful time period | Not applicable | Not applicable |
|  | d) | Consider plots to visualize results (e.g. forest plot, scatterplot of associations between genetic variants and outcome versus between genetic variants and exposure) | Figure 2, Figure 3, Table 1 and Table 2 | Figure 2, Figure 3, Table 1 and Table 2 |
| 12 | **Assessment of assumptions** |  |  |  |
|  | a) | Report the assessment of the validity of the assumptions | 6-8 | Other sensitive analyses method as maximum likelihood, weighted median also detected the same results (Supplementary Table 3 and 4). The MR-Egger regression analysis showed that there was no horizontal pleiotropy (Egger intercept cognitive performance = -0.0002, *p* = 0.91; Egger intercept FIS = -0.001, *p* = 0.87) (Supplementary Table 6). Horizontal pleiotropy was shown by the results of the MR-PRESSO analysis, which also identified some outliers (Supplementary Table 7). The results also indicated that, after removing this SNP, trends of recalculate results are consistent with the initial results (IVW cognitive performance, OR = 1.62, 95% CI = 1.23-2.14, *p* = 1.29E-03; IVW FIS, OR = 3.58, 95% CI = 1.87-6.84, *p* = 3.72E-04). Further leave-one-out tests suggested that the associations between glucosamine and FIS was reliable, while the association between glucosamine and cognitive performance was less robust (Supplementary Figure 1). |
|  | b) | Report any additional statistics (e.g., assessments of heterogeneity across genetic variants, such as *I^2^*, Q statistic or E-value) | 6-8 | The main analytical method applied in the MR analysis, IVW-FE discovered that taking glucosamine significantly increased cognitive performance (OR = 1.40, 95% CI = 1.15-1.70, *p* = 8.46E-04), and FIS (OR = 2.50, CI = 1.47-4.26, *p* = 7.50E-04) (Figure 2, Supplementary Table 3 and 4). Besides, due to the detected some heterogeneity by Cochran Q statistic (*p* < 0.01), we applied the IVW-MRE method and also detected consistent results (Supplementary Table 3, 4 and 5). Other sensitive analyses method as maximum likelihood, weighted median also detected the same results (Supplementary Table 3 and 4). The MR-Egger regression analysis showed that there was no horizontal pleiotropy (Egger intercept cognitive performance = -0.0002, *p* = 0.91; Egger intercept FIS = -0.001, *p* = 0.87) (Supplementary Table 6). Horizontal pleiotropy was shown by the results of the MR-PRESSO analysis, which also identified some outliers (Supplementary Table 7). The results also indicated that, after removing this SNP, trends of recalculate results are consistent with the initial results (IVW cognitive performance, OR = 1.62, 95% CI = 1.23-2.14, *p* = 1.29E-03; IVW FIS, OR = 3.58, 95% CI = 1.87-6.84, *p* = 3.72E-04). The IVW-FE method showed that glucosamine was associated with increased ALM (OR = 1.44, 95% CI = 1.26-1.65, *p* = 6.45E-08), WBFM (OR = 1.15, 95% CI = 1.05-1.25, *p* = 1.97E-03), usual walking pace (OR = 1.26, 95% CI = 1.16-1.38, *p* = 2.55E-07) as well as moderate to vigorous physical activity levels (OR = 1.26, 95% CI = 1.08-1.47, *p* = 3.29E-03), but associated with decreased FA risk (OR = 0.85, 95% CI = 0.79-0.92, *p* = 3.77E-05) and decreased DNA methylation GrimAge acceleration (OR = 0.02, 95% CI = 0.002-0.21, *p* = 0.001) (Figure 2, Supplementary Table 3 and 4). Besides, there was no significant causal associations between glucosamine and low hand grip strength (OR = 1.17, 95% CI = 0.69-1.97, *p* = 0.56). Besides, due to the detected some heterogeneity by Cochran Q statistic (*p* < 0.01), the IVW-MRE method also detected consistently effective effect of glucosamine on FA, physical activity and usual walking pace (Supplementary Table 3, 4 and 5). The MR-Egger regression analysis showed that there was no horizontal pleiotropy but except between glucosamine and physical activity (Egger intercept = -0.003, *p* = 0.03) (Supplementary Table 6). Horizontal pleiotropy was shown by the results of the MR-PRESSO analysis, which also identified some outliers (Supplementary Table 6). The results also indicated that, after removing this SNP, trends of recalculate results are consistent with the initial results. |
| 13 | **Sensitivity analyses and additional analyses** |  | 6-8 |  |
|  | a) | Report any sensitivity analyses to assess the robustness of the main results to violations of the assumptions | 6-8 | The main analytical method applied in the MR analysis, IVW-FE discovered that taking glucosamine significantly increased cognitive performance (OR = 1.40, 95% CI = 1.15-1.70, *p* = 8.46E-04), and FIS (OR = 2.50, CI = 1.47-4.26, *p* = 7.50E-04) (Figure 2, Supplementary Table 3 and 4). Besides, due to the detected some heterogeneity by Cochran Q statistic (*p* < 0.01), we applied the IVW-MRE method and also detected consistent results (Supplementary Table 3, 4 and 5). Other sensitive analyses method as maximum likelihood, weighted median also detected the same results (Supplementary Table 3 and 4). The MR-Egger regression analysis showed that there was no horizontal pleiotropy (Egger intercept cognitive performance = -0.0002, *p*= 0.91; Egger intercept FIS = -0.001, *p*= 0.87) (Supplementary Table 6). Horizontal pleiotropy was shown by the results of the MR-PRESSO analysis, which also identified some outliers (Supplementary Table 7). The results also indicated that, after removing this SNP, trends of recalculate results are consistent with the initial results (IVW cognitive performance, OR = 1.62, 95% CI = 1.23-2.14, *p* = 1.29E-03; IVW FIS, OR = 3.58, 95% CI = 1.87-6.84, *p* = 3.72E-04). The IVW-FE method showed that glucosamine was associated with increased ALM (OR = 1.44, 95% CI = 1.26-1.65, *p* = 6.45E-08), WBFM (OR = 1.15, 95% CI = 1.05-1.25, *p* = 1.97E-03), usual walking pace (OR = 1.26, 95% CI = 1.16-1.38, *p* = 2.55E-07) as well as moderate to vigorous physical activity levels (OR = 1.26, 95% CI = 1.08-1.47, *p* = 3.29E-03), but associated with decreased FA risk (OR = 0.85, 95% CI = 0.79-0.92, *p* = 3.77E-05) and decreased DNA methylation GrimAge acceleration (OR = 0.02, 95% CI = 0.002-0.21, *p* = 0.001) (Figure 2, Supplementary Table 3 and 4). Besides, there was no significant causal associations between glucosamine and low hand grip strength (OR = 1.17, 95% CI = 0.69-1.97, *p* = 0.56). Besides, due to the detected some heterogeneity by Cochran Q statistic (p < 0.01), the IVW-MRE method also detected consistently effective effect of glucosamine on FA, physical activity and usual walking pace (Supplementary Table 3, 4 and 5). The MR-Egger regression analysis showed that there was no horizontal pleiotropy but except between glucosamine and physical activity (Egger intercept = -0.003, *p* = 0.03) (Supplementary Table 6). Horizontal pleiotropy was shown by the results of the MR-PRESSO analysis, which also identified some outliers (Supplementary Table 6). The results also indicated that, after removing this SNP, trends of recalculate results are consistent with the initial results. |
|  | b) | Report results from other sensitivity analyses or additional analyses | 6-8 | The main analytical method applied in the MR analysis, IVW-FE discovered that taking glucosamine significantly increased cognitive performance (OR = 1.40, 95% CI = 1.15-1.70, *p* = 8.46E-04), and FIS (OR = 2.50, CI = 1.47-4.26, *p* = 7.50E-04) (Figure 2, Supplementary Table 3 and 4). Besides, due to the detected some heterogeneity by Cochran Q statistic (*p* < 0.01), we applied the IVW-MRE method and also detected consistent results (Supplementary Table 3, 4 and 5). Other sensitive analyses method as maximum likelihood, weighted median also detected the same results (Supplementary Table 3 and 4). The MR-Egger regression analysis showed that there was no horizontal pleiotropy (Egger intercept cognitive performance = -0.0002, *p* = 0.91; Egger intercept FIS = -0.001, *p* = 0.87) (Supplementary Table 6). Horizontal pleiotropy was shown by the results of the MR-PRESSO analysis, which also identified some outliers (Supplementary Table 7). The results also indicated that, after removing this SNP, trends of recalculate results are consistent with the initial results (IVW cognitive performance, OR = 1.62, 95% CI = 1.23-2.14, *p* = 1.29E-03; IVW FIS, OR = 3.58, 95% CI = 1.87-6.84, *p* = 3.72E-04). The IVW-FE method showed that glucosamine was associated with increased ALM (OR = 1.44, 95% CI = 1.26-1.65, *p* = 6.45E-08), WBFM (OR = 1.15, 95% CI = 1.05-1.25, *p* = 1.97E-03), usual walking pace (OR = 1.26, 95% CI = 1.16-1.38, p = 2.55E-07) as well as moderate to vigorous physical activity levels (OR = 1.26, 95% CI = 1.08-1.47, *p* = 3.29E-03), but associated with decreased FA risk (OR = 0.85, 95% CI = 0.79-0.92, *p* = 3.77E-05) and decreased DNA methylation GrimAge acceleration (OR = 0.02, 95% CI = 0.002-0.21, *p* = 0.001) (Figure 2, Supplementary Table 3 and 4). Besides, there was no significant causal associations between glucosamine and low hand grip strength (OR = 1.17, 95% CI = 0.69-1.97, *p* = 0.56). Besides, due to the detected some heterogeneity by Cochran Q statistic (*p* < 0.01), the IVW-MRE method also detected consistently effective effect of glucosamine on FA, physical activity and usual walking pace (Supplementary Table 3, 4 and 5). The MR-Egger regression analysis showed that there was no horizontal pleiotropy but except between glucosamine and physical activity (Egger intercept = -0.003, *p* = 0.03) (Supplementary Table 6). Horizontal pleiotropy was shown by the results of the MR-PRESSO analysis, which also identified some outliers (Supplementary Table 6). The results also indicated that, after removing this SNP, trends of recalculate results are consistent with the initial results. |
|  | c) | Report any assessment of direction of causal relationship (e.g., bidirectional MR) | Not applicable | Not applicable |
|  | d) | When relevant, report and compare with estimates from non-MR analyses | Not applicable | Not applicable |
|  | e) | Consider additional plots to visualize results (e.g., leave-one-out analyses) | Supplementary Figure 1 and Supplementary Figure 2 | Further leave-one-out tests suggested that the associations between glucosamine and FIS was reliable, while the association between glucosamine and cognitive performance was less robust (Supplementary Figure 1).  Further leave-one-out tests suggested that the associations were reliable, while the association between glucosamine and ALM was less robust (Supplementary Figure 2). |
|  | **DISCUSSION** |  |  |  |
| 14 | **Key results** | Summarize key results with reference to study objectives | 8 | In this research, we primarily used large-sample GWAS data to assess the connections between glucosamine and cognition as well as sarcopenia proxy indicators by MR analysis. We conducted various MR analysis methods including UVMR, MVMR and mediation analysis, which showed that (1) glucosamine use is probably associated with enhanced cognitive performance, FIS, ALM, WBFM, usual walking pace and physical activity, and decreased risk of FA and DNA methylation GrimAge acceleration; (2) this protective effect for glucosamine on cognitive performance, FIS, ALM, WBFM, usual walking pace and physical activity remain even after adjusting for both OA and BMI; (3) CRP may mediate the association of glucosamine and ALM, WBFM, usual walking pace, and physical activity; (4) BMR may mediate the association of glucosamine and cognitive performance, FIS, ALM, WBFM, and usual walking pace. |
| 15 | **Limitations** | Discuss limitations of the study, taking into account the validity of the IV assumptions, other sources of potential bias, and imprecision. Discuss both direction and magnitude of any potential bias and any efforts to address them | 9 | Although our research has a substantial sample size, it is susceptible to many constraints. Primarily, the data for all participants in this research mostly originated from European nations, thereby limiting the generalizability of the findings to other ethnic contexts. Secondly, because to the use of summary-level statistics in the published data, we were unable to examine a non-linear causal relationship between glucosamine and indicators of cognition and sarcopenia. Subsequent investigations should prioritize this issue. |
| 16 | **Interpretation** |  |  |  |
|  | a) | Meaning: Give a cautious overall interpretation of results in the context of their limitations and in comparison with other studies | 8-9 | A cross-sectional study based on UK Biobank participants aged 37-73 years demonstrated that glucosamine use exhibited a positive effect on cognitive function. Longitudinal research including 214,945 people during a median follow-up of 12 years, found a substantial inverse association between regular usage of glucosamine and the occurrence of vascular dementia in older individuals.  A recent MR study found that lifelong higher levels of glucosamine may increase life expectancy.  Furthermore, it is important to acknowledge that MR analysis provides less compelling evidence for causality compared to RCTs. Therefore, additional high-quality RCT evidence is still required to complement and reinforce our findings. |
|  | b) | Mechanism: Discuss underlying biological mechanisms that could drive a potential causal relationship between the investigated exposure and the outcome, and whether the gene-environment equivalence assumption is reasonable. Use causal language carefully, clarifying that IV estimates may provide causal effects only under certain assumptions | 8-9 | Longitudinal research including 214,945 people during a median follow-up of 12 years, found a substantial inverse association between regular usage of glucosamine and the occurrence of vascular dementia in older individuals.  Glucosamine may prevent bone loss in aging and adjust the bone turnover. A double-blinded, randomized controlled trial revealed that the administration of glucosamine during a 12-week strength-training program resulted in enhanced maximal muscle strength gain in patients with OA, when compared to treatment with a placebo. Besides, a randomized, double-blind, placebo-controlled study in Japan investigated that consumption of a combination of milk-fat globule membrane and glucosamine may improve joint function and physical performance. Cerbo et al study reported that the dietary supplement including glucosamine could improve facial photoaging and skin sebum, hydration and tonicity, which may be by modulating serum fibronectin, neutrophil elastase 2, hyaluronic acid and carbonylated proteins. The protective effect may due to that glucosamine significantly increased brain-derived neurotrophic factor (BDNF) levels and induced cAMP responsive element reporter activity. |
|  | c) | Clinical relevance: Discuss whether the results have clinical or public policy relevance, and to what extent they inform effect sizes of possible interventions | 8-9 | These results are consistent with us as we found that genetically predicted glucosamine use causally associated with enhanced ALM, WBFM, usual walking pace and physical activity, and decreased risk of FA. In addition, a recent MR study found that lifelong higher levels of glucosamine may increase life expectancy. |
| 17 | **Generalizability** | Discuss the generalizability of the study results (a) to other populations, (b) across other exposure periods/timings, and (c) across other levels of exposure | 9 | Although our research has a substantial sample size, it is susceptible to many constraints. Primarily, the data for all participants in this research mostly originated from European nations, thereby limiting the generalizability of the findings to other ethnic contexts. |
|  | **OTHER INFORMATION** |  |  |  |
| 18 | **Funding** | Describe sources of funding and the role of funders in the present study and, if applicable, sources of funding for the databases and original study or studies on which the present study is based | 10-11 | This study was supported by the National Natural Science Foundation of China (NO. 82301371 to Y.T.), Post-Doctor Research Project, West China Hospital, Sichuan University (No.2021HXBH059 to Y.T.), the Fundamental Research Funds for the Central Universities (No. 2023SCU12075 to Y.T.), the Science and Technology Plan Project of Sichuan Province (No. 2023NSFSC1780 to Y.T.), and the Key R&D projects of Sichuan Science and Technology Plan (No. 23ZDYF1157 to G.C.). |
| 19 | **Data and data sharing** | Provide the data used to perform all analyses or report where and how the data can be accessed, and reference these sources in the article. Provide the statistical code needed to reproduce the results in the article, or report whether the code is publicly accessible and if so, where | 10 | The datasets supporting the conclusions of this article are available in the IEU Open GWAS (https://gwas.mrcieu.ac.uk/). |
| 20 | **Conflicts of Interest** | All authors should declare all potential conflicts of interest | 10 | The authors declare that the research was conducted in the absence of any commercial or financial relationships that could be construed as a potential conflict of interest. |

This checklist is copyrighted by the Equator Network under the Creative Commons Attribution 3.0 Unported (CC BY 3.0) license.

1. Skrivankova VW, Richmond RC, Woolf BAR, Yarmolinsky J, Davies NM, Swanson SA, et al. Strengthening the Reporting of Observational Studies in Epidemiology using Mendelian Randomization (STROBE-MR) Statement. JAMA. 2021;under review.

2. Skrivankova VW, Richmond RC, Woolf BAR, Davies NM, Swanson SA, VanderWeele TJ, et al. Strengthening the Reporting of Observational Studies in Epidemiology using Mendelian Randomisation (STROBE-MR): Explanation and Elaboration. BMJ. 2021;375:n2233.
