# Supplementary material for: Causality of genetically determined glucosamine supplementation on cognition and sarcopenia: a Mendelian randomization study
Source: Front Endocrinol (Lausanne). 2024 Dec 23;15:1404308. doi: 10.3389/fendo.2024.1404308 (PMC11700805; doi:10.3389/fendo.2024.1404308)
Supplement: Supplementary file 2 [file DataSheet2.docx]

Supplementary Material

# Supplementary Figures

## Supplementary Figure 1
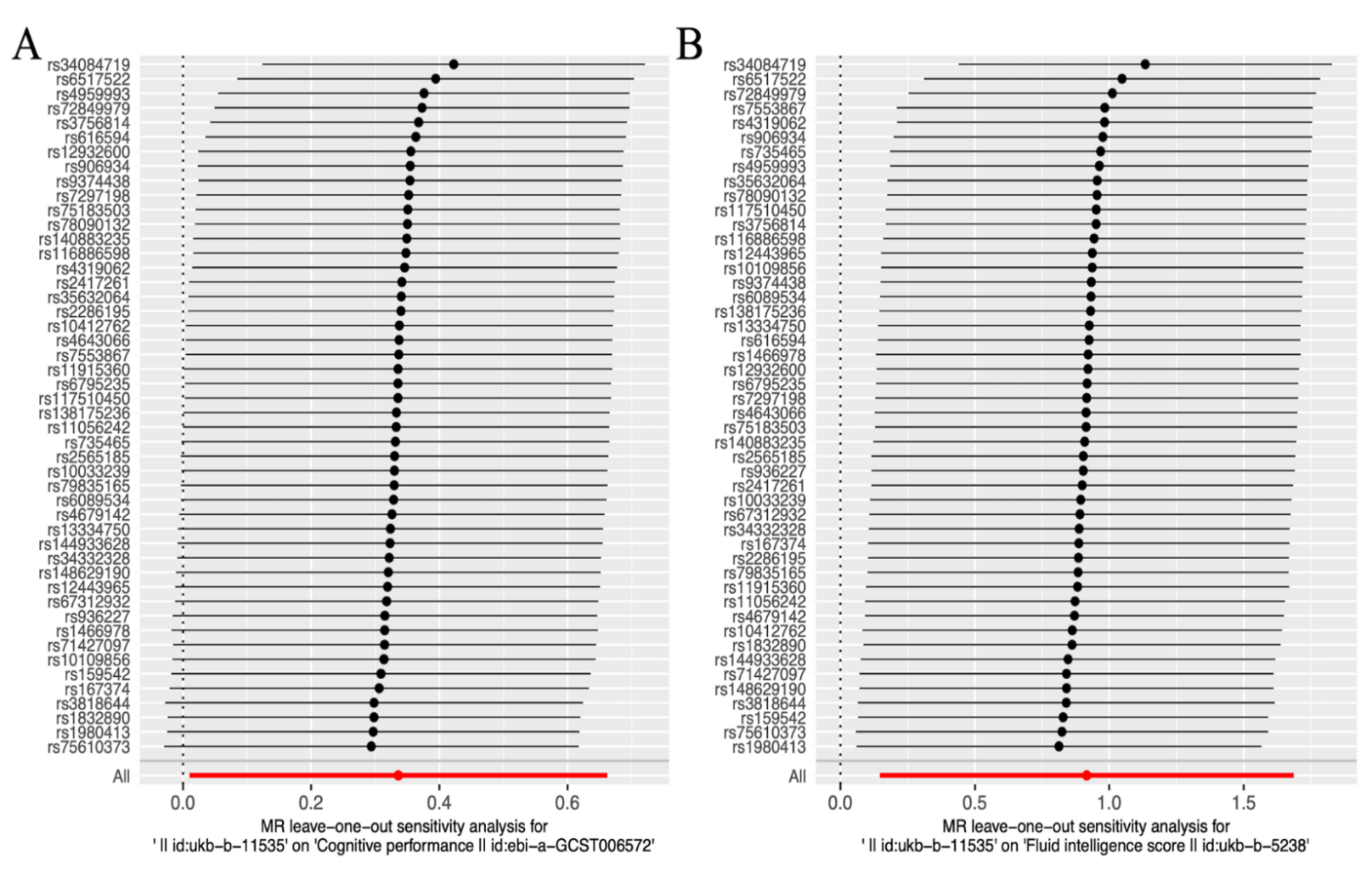


**Supplementary Figure 1.** Forest plots of leave-one-out for glucosamine on cognitive performance and FIS.

## Supplementary Figure 2


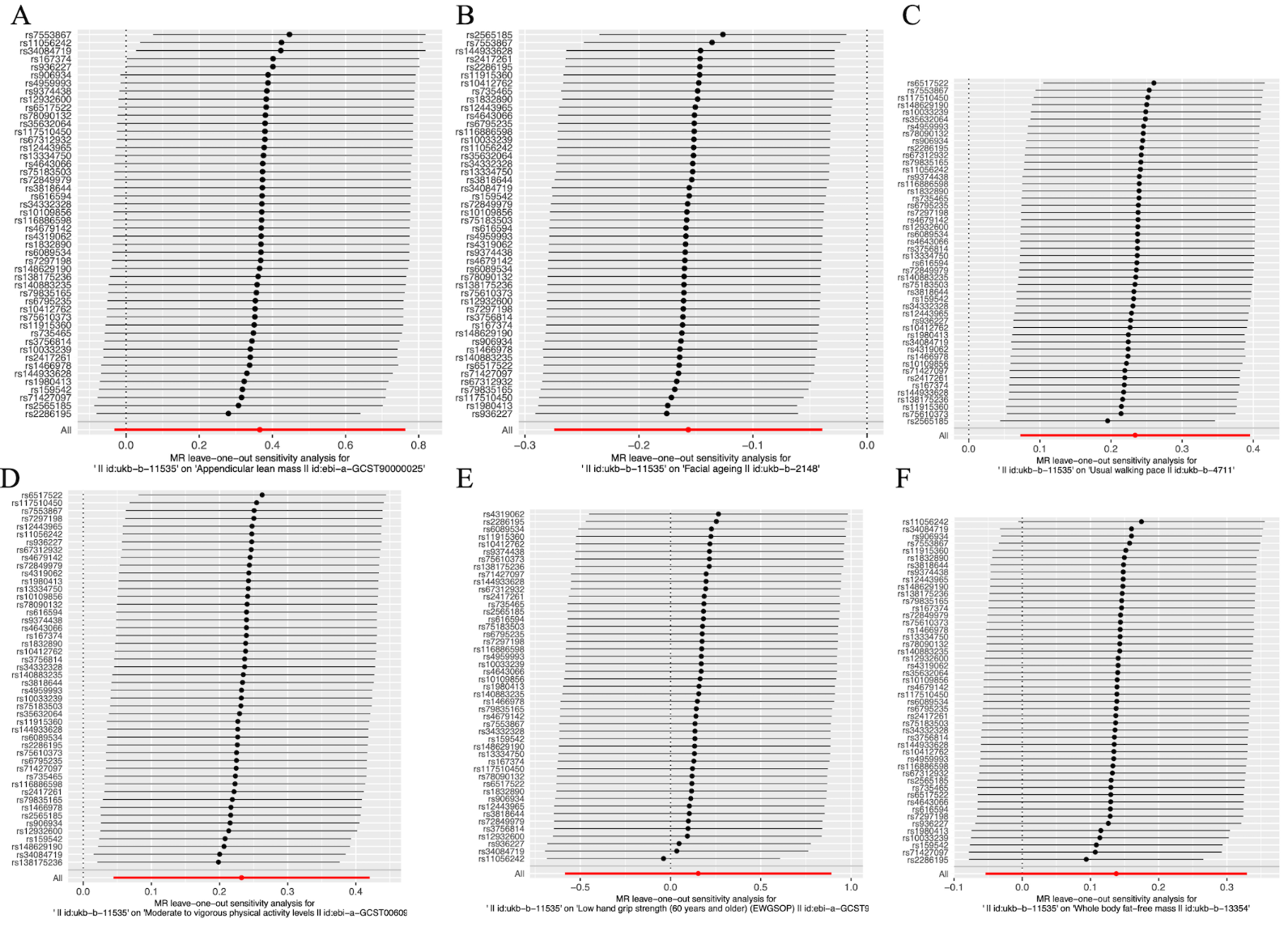


**Supplementary Figure 2.** Forest plots of leave-one-out for glucosamine on ALM, FA, usual walking pace, moderate to vigorous physical activity levels, low hand grip strength and WBFM.
